# Supplementary material for: Overestimated nitrogen loss from denitrification for natural terrestrial ecosystems in CMIP6 Earth System Models
Source: Nat Commun. 2023 May 27;14:3065. doi: 10.1038/s41467-023-38803-z (PMC10224944; doi:10.1038/s41467-023-38803-z)
Supplement: Supplementary file 1 — Supplementary Information [file 41467_2023_38803_MOESM1_ESM.pdf]

Supplementary Information for "Overestimated nitrogen loss from denitrification for natural terrestrial ecosystems in CMIP6 Earth System Models"

Maoyuan Feng[1,2], Shushi Peng[1,2*], Yilong Wang[3], Philippe Ciais[4,5], Daniel S. Goll[4], Jinfeng Chang[6], Yunting Fang[7], Benjamin Z. Houlton[8], Gang Liu[1,2], Yan Sun[9], Yi Xi[1,4]

[1]Sino-French Institute for Earth System Science, College of Urban and Environmental Sciences, and Laboratory for Earth Surface Processes, Peking University, Beijing, China

[2]Institute of Carbon Neutrality, Peking University, Beijing, China

[3]Key Laboratory of Alpine Ecology, Institute of Tibetan Plateau Research, Chinese Academy of Sciences, Beijing, China

[4]Laboratoire des Sciences du Climat et de l'Environnement, LSCE/IPSL, CEA-CNRS-UVSQ, Université Paris-Saclay, Gif-sur-Yvette, France

[5]The Cyprus Institute 20 Konstantinou Kavafi Street, 2121, Nicosia, Cyprus

[6]College of Environmental and Resource Sciences, Zhejiang University, Hangzhou, China

[7]CAS Key Laboratory of Forest Ecology and Management, Institute of Applied Ecology, Chinese Academy of Sciences, Shenyang, China

[8]Department of Ecology and Evolutionary Biology and Department of Global Development, CALS, Cornell University, Ithaca, NY, USA

[9]College of Marine Life Sciences, Ocean University of China, Qingdao, China

*Correspondence to Shushi Peng (speng@pku.edu.cn)

Table of Contents

1. Random Forest algorithm to derive global soil d15N map 3

2. The isotope-benchmarking based f_{denit} and denitrification N losses 5

3. The sensitivity of the isotope-based f_{denit} and denitrification N loss to the isotope effect of denitrification 6

4. Impacts of NH_3 volatilization on f_{denit} 9

5. The representations of N losses in different CMIP6 ESMs 11

6. Evaluation of the effects of the terrestrial N sink on isotope-based f_{denit} 12

References 53

1. Random Forest algorithm to derive global soil $\delta^{15}N$ map

We aggregated 5887 site-level direct measurements of soil $\delta^{15}N$ in natural ecosystems[1,2] into 955 0.1°×0.1° grid cells (locations shown in Supplementary Fig. 1). We excluded those grid cells for which any of the 16 predictors have missing or invalid values, and used the soil $\delta^{15}N$ observations in the remaining 933 0.1°×0.1° grid cells to train the Random Forest (RF) algorithm. Specifically, this training involved adopting 500 decision trees using a well-established Python v3.8.5 package, RandomForestRegressor. For each decision tree, the bootstrap strategy was activated for sampling the predictors, the maximum feature was set to be the square root of feature numbers (i.e., 4 features), and the minimum leaf of the decision tree was set to 1. This well-trained RF model performs well in capturing the relationship between 16 predictors and soil $\delta^{15}N$ (R^2=0.92, Root Mean Square Error (RMSE)=0.77‰), and in predicting the soil $\delta^{15}N$ (R^2=0.55, RMSE=1.83‰) (Supplementary Fig. 2). To examine the reliability of the RF model, we further conducted a K-fold (K=10) cross validation by training the RF model with 90% of the samples and testing it with the remaining 10% of the samples. The training and testing processes were repeated 100 times, resulting in the frequency distribution of the variance explained by the RF model as shown in Supplementary Fig. 3. We found that withholding 10% of samples would only slightly degenerate the model performance, indicating that the RF model predictions of soil $\delta^{15}N$ would be robust.

By applying the well-trained RF model to the global scale, we obtained two similar global maps of soil $\delta^{15}N$ (Supplementary Fig. 4), corresponding to RF models with N depositions (NH_x, NO_y; two of the 16 predictors) from Tian *et al.*[3] and European Monitoring and Evaluation Programme (EMEP)[4], respectively. By using six sets of global maps of N inputs as weights, we estimated the ensemble mean of global soil $\delta^{15}N$ as 4.82‰ (Supplementary Table 1), slightly lower than a

previous estimate of 5.5‰ by Houlton *et al.*[5]. These two global maps of soil $\delta^{15}N$ have similar spatial distributions. Taking Supplementary Fig. 4a as an example, the global soil $\delta^{15}N$ map has maximum and minimum latitudinal means of 7.1‰ and 2.5‰, respectively, resulting in a latitudinal gradient of -0.5‰ per 10° increase in the latitude. Supplementary Fig. 4b and Fig. 4d illustrate the uncertainty, or SD, of the soil $\delta^{15}N$ values, with 90% of the grid cells having a SD smaller than 3.2‰.

Compared to the global soil $\delta^{15}N$ map in Amundson *et al.*[6], our global soil $\delta^{15}N$ map has an increased value of R^2 between observations and predictions across 933 grid cells (from 0.20 to 0.93) and a decreased RMSE (from 2.82‰ to 0.77‰) (Supplementary Fig. 5). Specifically, compared to Amundson *et al.*[6], our global map has higher $\delta^{15}N$ signals in the Amazonia and has lower $\delta^{15}N$ signals in Southeast and East Asia. Amundson *et al.*[6] collected very limited soil $\delta^{15}N$ observations (~50) at 6 sites (4-6 elevations at each site), while we have utilized thousands of observations covering different climate zones, vegetation types and six continents. Thus, we believed that our map has higher confidence than the predicted map in Amundson *et al.*[6]. (Supplementary Fig. 1).

The RF model identified N fixing bacteria (Nfixer), temperature (T), Ectomycorrhizal fungi (ECM), aridity index (P/PET), NO_y deposition and Arbuscular mycorrhizal fungi (AM) as the six leading predictors in producing global map of soil $\delta^{15}N$ (Supplementary Figs. 17–19). We illustrated the linear regression relationship between soil $\delta^{15}N$ observations and these six leading predictors (Supplementary Fig. 18), and also showed the nonlinear partial dependence relationship between RF-predicted soil $\delta^{15}N$ and these six predictors (Supplementary Fig. 19). By comparing the linear regression relationships with previous works[1,6], these outputs confirmed the significant role of temperature in predicting global soil $\delta^{15}N$, as was the case in the (piecewise) linear regression

model of Craine *et al.*[1] (Supplementary Figs. 20–21) and bivariate linear regression model of Amundson *et al.*[6], but suggested that Amundson *et al.*[6] overestimated the role of the precipitation over the other soil and microbial predictors. Moreover, we would like to highlight the crucial roles of microsymbionts (Nfixer, ECM) and N deposition, which are potential variables for improving model representations of the N cycle considering the N isotope benchmark. Particularly, the crucial roles of microbial symbionts result from that the N fixing bacteria assimilates atmospheric N_2 into soil with its $\delta^{15}N$ signal close to zero, and the plants associated with ECM and AM have different pathways of N uptake from soil, with the isotope fractionation higher for ECM than AM[12,14].

2. The isotope-benchmarking based f_{denit} and denitrification N losses

The isotope-benchmarking based f_{denit} depends on the relative fractions of N inputs from rock N weathering, N deposition, and biological N fixation (BNF) as well as their $\delta^{15}N$ signals. To account for the uncertainty in these N inputs, we used six sets of global N inputs as well as their $\delta^{15}N$ signals by combining one global map of rock N weathering from Houlton *et al.*[7] (10 Tg N yr^{-1} with a global mean $\delta^{15}N$ of 4.02‰), two global maps of N deposition from Tian *et al.*[3] and EMEP[4] (an average of 40 Tg N yr^{-1} with a constant $\delta^{15}N$ of 0‰) and three global maps of BNF from Peng *et al.*[8] (an average of 57 Tg N yr^{-1} with a constant $\delta^{15}N$ of -2‰). For these six sets of global maps, the global N input ranges from 92 to 133 Tg N yr^{-1} while the global mean of $\delta^{15}N_{input}$ ranges from -0.94‰ to -0.50‰ (Supplementary Table 1). With global maps of soil $\delta^{15}N$ and N input $\delta^{15}N$, we derived an ensemble of global maps of isotope-based f_{denit} that have similar spatial patterns (Supplementary Fig. 6), with their global means ranging from 0.41 to 0.43 (Supplementary Table 1). We adopted the mean of these six global maps of f_{denit} as the isotope-benchmarking based one, which has a global mean of 0.42. Moreover, we found that the uncertainty (quantified by standard deviations,

SDs) of the isotope-based f_{denit} across six ensembles (Supplementary Fig. 8b) is much lower than the uncertainty from upscaling f_{denit} from soil $\delta^{15}N$ (Supplementary Fig. 8a), where the SD in 90% of the grid cells is less than 0.22.

We compared the global map of the isotope-benchmarking based f_{denit} derived in this study with the map derived from the global soil $\delta^{15}N$ map of Amundson *et al.*[6], and that presented in Goll *et al.*[9] (Supplementary Fig. 7). Our global map of isotope-benchmarking based f_{denit} (Supplementary Fig. 7a) is similar to the global map of isotope-based f_{denit} from Amundson *et al.*[6] (Supplementary Fig. 7b) but has a larger latitudinal gradient. This difference results from the higher values of soil $\delta^{15}N$ observations in the tropics presented in the new $\delta^{15}N$ datasets. However, the global map of f_{denit} obtained from the JSBACH model (Supplementary Fig. 7c) shows relatively lower f_{denit} in the tropics (Amazonia, northern Africa, and southeast Asia) but relatively higher values in the temperate regions (southwest U.S., southern Africa, Australia), which is contradictory to our common knowledge that the tropics has a higher fraction of N loss from the denitrification pathway. This finding highlights the urgent need to improve the representation of denitrification in land surface models.

With the isotope-benchmarking based f_{denit}, we estimated the steady-state denitrification N loss by assuming the total N loss to be equal to the total N input. We obtained six similar global maps of steady-state denitrification N loss corresponding to the six sets of global maps of N inputs and isotope-based f_{denit} (Supplementary Fig. 15). The ensemble mean of the denitrification N loss was estimated at 45±9 Tg N yr^{-1}, with the maximum and minimum values being 38 and 57 Tg N yr^{-1}, respectively (Supplementary Table 1).

3. The sensitivity of the isotope-based f_{denit} and denitrification N loss to the isotope effect of denitrification

We examined the sensitivity of the isotope-based f_{denit} and the resulting denitrification N loss to the isotope effect of denitrification (ε_{denit}), or the so-called fractionation factor, by adopting various global constant values and two temperature-inferred fields (Supplementary Table 2; Supplementary Fig. 10). The isotope effect of denitrification (ε_{denit}) has been reported to have large variations, e.g., 31–65‰ has been measured in laboratory incubation[10–12], while 10–20‰ has been observed in natural soil communities[13–15]. Since the soil $\delta^{15}N$ observations used in this study were collected in natural soil, we tested the sensitivity of the isotope-based f_{denit} to ε_{denit} by varying ε_{denit} from 10‰ to 20‰ (i.e., 10‰, 13‰, 16‰, 20‰), where 13‰ and 16‰ are the two most widely adopted levels in previous publications[5,13,16,17]. Note that we tested the sensitivity using only one out of the six global N input maps, i.e., the one with a global N input of 130 Tg N yr^{-1}. For global constant ε_{denit}, the global pattern of the isotope-based f_{denit} is primarily controlled by the global soil $\delta^{15}N$ map upscaled by the RF model. Medium (16‰) and high (20‰) levels of ε_{denit} result in global means of f_{denit} of 0.35 and 0.28, respectively (Supplementary Table 2), which are consistent with previous estimates[13,16]. Moreover, we found that the global mean of isotope-based f_{denit} decreases by ~0.03 with each 1‰ increase in ε_{denit} (Supplementary Fig. 10; Supplementary Table 2). The discrepancy between the isotope-benchmarking based f_{denit} and that simulated by the CMIP6 ESMs shown in Fig. 2 in the main would be enlarged by a higher ε_{denit}, i.e., a 1‰ increase in ε_{denit} would enlarge the discrepancy by ~13% (Supplementary Fig. 10; Supplementary Table 2).

We also tested two global maps of ε_{denit} inferred from the global gridded air temperature (T): (1) $\varepsilon_{denit} = g_1(T) = a + b/T$, based on laboratory experimental observations suggested by Mariotti *et al.*

[18]; (2) $\varepsilon_{denit} = g_2(T) = a + b/(T_{max} + T_{min} - T)$, based on the common knowledge that ε_{denit} increases as one moves from high- to low-latitudinal regions. In these two temperature-dependent scenarios, the parameters a and b were evaluated by setting the maximum and minimum values of ε_{denit} to 20‰ and 10‰, respectively. Compared to the global constant ε_{denit}, the scenario $\varepsilon_{denit} = g_1(T)$ increases f_{denit} in tropical regions but decreases it in boreal regions; whereas the scenario with $\varepsilon_{denit} = g_2(T)$ does the opposite, increasing f_{denit} in boreal regions but decreasing it in the tropics. With these two assumed global maps of ε_{denit}, we found that the global mean values of f_{denit} varies from 0.33 to 0.47 (Supplementary Table 2).

Since the estimation of denitrification N loss depends strongly on the isotope-based f_{denit} that is sensitive to ε_{denit}, we also tested the sensitivity of the steady-state denitrification N loss to ε_{denit}. By using one out of the six N inputs (130 Tg N yr^{-1}), we obtained a value for the minimum denitrification N loss of 37±2 Tg N yr^{-1}, corresponding to the minimum isotope-based f_{denit} and the maximum ε_{denit}. We also obtained the maximum denitrification N loss as 73±3 Tg N yr^{-1}, corresponding to the maximum isotope-based f_{denit} and the minimum ε_{denit}. We found that the denitrification N loss decreased by ~3.6 Tg N yr^{-1} for each 1‰ increase in ε_{denit}. Moreover, we also found that ε_{denit}, inferred from the global gridded air temperature, corresponds to medium levels of denitrification N losses in our sensitivity analysis; the denitrification N loss with $\varepsilon_{denit} = g_1(T)$ is 19 Tg N yr^{-1} higher than that with $\varepsilon_{denit} = g_2(T)$.

Since the rock $\delta^{15}N$ signal is reported to have large variations and could affect the isotope-based f_{denit} significantly, we conducted the above sensitivity analysis using three different global maps of rock $\delta^{15}N$ signal to test the effect of low, medium and high levels of the signal (Supplementary

Table 2; Supplementary Fig. 21). We found that the sensitivities of f_{denit} and denitrification N loss to the isotope effect of denitrification are very close under different rock $\delta^{15}N$ levels, as the changes in rock $\delta^{15}N$ levels change the isotope-based f_{denit} and denitrification N loss systematically. Compared to a medium rock $\delta^{15}N$ signal, adopting the lithology $\delta^{15}N$ signal at the lower bound increases the global mean value of f_{denit} systematically by 0.01, and denitrification N loss by 2 Tg N yr^{-1}. Adopting the rock $\delta^{15}N$ signal at the upper bound has the opposite effect, decreasing the global mean of f_{denit} systematically by 0.01, and denitrification N loss by 2 Tg N yr^{-1}.

4. Impacts of NH_3 volatilization on f_{denit}

Although in natural ecosystems the NH_3 volatilization accounts for only 2.4 Tg N yr^{-1} [19,20] (2–3% of total N losses, which equates to total N inputs, 92–133 Tg N yr^{-1}, under steady state conditions (Supplementary Table 1)), this small NH_3 flux could have a substantial effect on soil $\delta^{15}N$ and the isotope-based f_{denit} due to the very high fractionation effects (29–35‰)[12,21]. Moreover, in ESMs, separating the NH_3 volatilization from the gaseous N loss could affect the simulated f_{denit}. Therefore, we analyzed the impacts of NH_3 volatilization on the isotope-based f_{denit} and ESMs' simulated f_{denit} . Since NH_3 volatilization is not amongst the ESM outputs available in the CMIP6 database, this analysis was based on two assumptions: (1) NH_3 volatilization occurs mainly in grid cells with >50% of the area covered by crops and pasture, where the NH_4^--form of N deposition will be highly affected by the fertilizer application; (2) NH_3 volatilization occurs mainly in tropical grid cells,

considering that NH_3 volatilization is more likely to occur in N-rich regions. Making these two assumptions, we established four scenarios to analyze the impacts of NH_3 volatilization:

(1) **Scenario 1 (S1):** 1% of total N losses is in the form of NH_3 volatilization, in grid cells with >50% of the area covered by crops and pasture. With six sets of global N inputs and the steady state assumption, this scenario results in a NH_3 volatilization of 0.1–0.2 Tg N yr^{-1}.

(2) **Scenario 2 (S2):** 5% of total N losses is in the form of NH_3 volatilization, in grid cells with >50% of the area covered by crops and pasture. With six sets of global N inputs and the steady state assumption, this scenario results in a NH_3 volatilization of 0.6–0.9 Tg N yr^{-1}.

(3) **Scenario 3 (S3):** 1% of total N losses is in the form of NH_3 volatilization, in tropical grid cells (25°S–25°N). With six sets of global N inputs and the steady state assumption, this scenario results in a NH_3 volatilization of 0.5–0.6 Tg N yr^{-1}.

(4) **Scenario 4 (S4):** 3% of total N losses is in the form of NH_3 volatilization, in tropical grid cells (25°S–25°N). With six sets of global N inputs and the steady state assumption, this scenario results in a NH_3 volatilization of 1.4–1.9 Tg N yr^{-1}, close to the previous estimates[19].

For isotope-based f_{denit}, following Houlton *et al.*[5], we analyzed the impacts of NH_3 volatilization by first deducting $f_{vol}\varepsilon_{vol}$ from the global soil $\delta^{15}N$ map (with a 29‰ isotope effect), and then re-deriving the isotope-based f_{denit} with the revised global soil $\delta^{15}N$ map. For all ESMs, we analyzed the impacts of NH_3 volatilization by first deducting the assumed NH_3 fluxes from gaseous N losses, and then re-evaluating the ESMs' f_{denit}. We found that, for both the isotope-based f_{denit} and the ESMs' f_{denit}, a higher fraction of NH_3 volatilization corresponds to a lower f_{denit} (Supplementary Table 8). Within the same scenario, the decrease in the isotope-based f_{denit} is larger than the decrease in the

ESMs' f_{denit}. For the four scenarios, the largest decrease in the isotope-based f_{denit} was -0.029, i.e., 7% of the original value (0.44).

Since the denitrification N loss is estimated as the product of N input and the isotope-based f_{denit} that relies on the term $f_{vol}\varepsilon_{vol}$, we assessed the impacts of NH_3 volatilization (f_{vol}) on the estimation of denitrification N loss. Using one out of the six sets of N inputs (130 Tg N yr^{-1}), we found that accounting for NH_3 volatilization could reduce the estimated denitrification N loss by 0–4 Tg N yr^{-1}, 0%–7% lower than the original estimate (56±2 Tg N yr^{-1}) (Supplementary Table 9). This suggests that, despite the high fractionation effect of NH_3 volatilization, the impacts of this small NH_3 volatilization on the isotope-based f_{denit} and denitrification N losses are limited.

5. The representations of N losses in different CMIP6 ESMs

We examined the mathematical representations of denitrification and leaching N losses in the CMIP6 ESMs (Supplementary Table 3). The representations of leaching N losses are similar across the 15 ESMs, with all of them using functions of the runoff and mineral N concentration. However, their representations of denitrification vary and can be grouped into two categories:

(1) Type I: Mineralization rate-based representation. The denitrification rate is formulated as a fraction of gross or net mineralization rate, which is also regulated by the environmental drivers such as temperature and soil moisture. Across the eight families of ESMs (15 ESMs), three families of ESMs have a mineralization rate-based representation of denitrification: (a) the EC-Earth3 family which uses the LPJ-GUESS land surface model; (b) UKESM1-0-LL with the JULES-ES-1.0 land surface model; and (c) ACCESS-ESM1-5 with the CASA-CNP land surface model. In (a), the denitrification N rate is represented as a function of the gross mineralization rate, whereas in (b) and (c), it is represented as a function of the net mineralization rate.

(2) Type II: Mineral pool-based representation. In these models the denitrification rate is formulated as the product of the mineral N pool and scaling factors driven by environmental variables such as temperature and soil moisture. Five of the eight families of ESMs have a mineral-pool based representation, and these can be classified into three groups: (a) the CESM2, NorESM2, and AWI-ESM families, which use the CLM5/4.5 land surface model; (b) the MPI-ESM family, with the JSBACH land surface model; and (c) MIROC-ES2L which uses the VISIT-e land surface model.

6. Evaluation of the effects of the terrestrial N sink on isotope-based f_{denit}

We utilized a box model for the plant-soil system to evaluate the effects of the terrestrial N sink on the soil $\delta^{15}N$ and the isotope-based f_{denit} (Supplementary Fig. 16). The box model consists of plant and soil N pools and the associated N fluxes, i.e., input, denitrification and leaching N losses, plant uptake and the return N fluxes. First, we derived all N fluxes and the corresponding $\delta^{15}N$ signals for the box model at steady state (Supplementary Fig. 16a). Specifically, we adopted a value for the total N loss and the total N input of 133 Tg N yr^{-1}, one out of the six sets of N inputs used in this study (Supplementary Table 1). By using the isotope balance equation (Eq. (1) in Methods), we estimated the soil $\delta^{15}N$ signal as 4.59‰. For the N fluxes between plant and soil, we estimated the N uptake as 778 Tg N yr^{-1} (with GPP from Keenan *et al.*[22], C:N ratio estimated from leaf N concentration produced by Moreno-Martínez *et al.*[23], and resorption coefficients from Deng *et al.*[24]), which is very close to the N uptake simulated by the Fixation and Uptake of Nitrogen (FUN) v2.0 model (777 Tg N yr^{-1})[25]. Based on the isotope balance in the soil, we derived the $\delta^{15}N$ signal of N uptake flux as -3.41‰. Under steady state conditions, the N return flux from plant to soil and its $\delta^{15}N$ signal are equal to those of N uptake (i.e., 778 Tg N yr^{-1} and -3.41‰).

Next, we modified the N fluxes in the box model to account for the terrestrial N sink under non-steady state conditions (Supplementary Fig. 16b and Fig. 16c). The terrestrial N sink was simulated as 25±7 Tg N yr^{-1} by the CMIP6 ESMs, with its maximum and minimum values of 44 and 18 Tg N yr^{-1}, respectively (Supplementary Table 5). Since a larger terrestrial N sink is expected to have a larger effect on the isotope-based f_{denit}, we adopted the mean (25 Tg N yr^{-1}) and maximum (44 Tg N yr^{-1}) of the terrestrial N sink in the following analysis. As shown in Supplementary Fig. 16b, we increased the plant N uptake by 25 Tg N yr^{-1} and decreased the total N loss by 25 Tg N yr^{-1}, such that the plant-soil system had an N sink of 25 Tg N yr^{-1}. Supplementary Fig. 16c illustrates how we increased the plant N uptake by 44 Tg N yr^{-1} and decreased the total N loss by 44 Tg N yr^{-1}, such that the plant-soil system had an N sink of 44 Tg N yr^{-1}. For the non-steady state, we used the Eulerian method to assess the effect of the terrestrial N sink on soil $\delta^{15}N$, with the dynamics of soil $\delta^{15}N$ formulated as follows:

$$d(N_{soil}\delta^{15}N_{soil})/dt = I\cdot\delta^{15}N_I + R\cdot\delta^{15}N_R - D\cdot\delta^{15}N_D - L\cdot\delta^{15}N_L - U\cdot\delta^{15}N_U \quad (1)$$

where N_{soil} is the N stock in the soil pool, and *I*, *D*, *L*, *U*, and *R* are the input, denitrification, leaching, uptake and return N fluxes, respectively. $\delta^{15}N_{soil}$ is the soil $\delta^{15}N$, and $\delta^{15}N_I$, $\delta^{15}N_D$, $\delta^{15}N_L$, $\delta^{15}N_U$, and $\delta^{15}N_R$ are $\delta^{15}N$ signals for the input, denitrification, leaching, uptake and return N fluxes, respectively. By simulating dynamical variations of soil $\delta^{15}N$ for 50 years (a time period representing the sharply disturbed N cycle, 1960–2010 or recent decades[3]), we obtained soil $\delta^{15}N$ as 4.61‰ and 4.63‰ for the cases with terrestrial N sinks of 25 and 44 Tg N yr^{-1}, respectively. This suggests that an N sink of 25 Tg N yr^{-1} could increase the soil $\delta^{15}N$ by only 0.02‰ (Supplementary Fig. 16b), and that an N sink of 44 Tg N yr^{-1} could increase the soil $\delta^{15}N$ by 0.04‰ (Supplementary Fig. 16c).

Finally, with the use of Eq. (2) in Methods, we evaluated the bias in the derived isotope-based f_{denit} by considering the soil $\delta^{15}N$ differences under steady and non-steady states. The results show that a terrestrial N sink of 25 Tg N yr^{-1} results in a 0.002 increase in the isotope-based f_{denit} (Supplementary Fig. 16b), while a terrestrial N sink of 44 Tg N yr^{-1} results in a 0.004 increase (Supplementary Fig. 16c). Overall, the terrestrial N sink could, at most, results in a <2% (0.004/0.42=1%) bias in the derivation of isotope-benchmarking based f_{denit}. Thus, the isotope-benchmarking based f_{denit} derived at steady state can be safely used to approximate non-steady state conditions without causing any significant bias in the estimation of denitrification N loss.

Supplementary Figures

Supplementary Figure 1. Locations of soil $\delta^{15}N$ measurements (n=956 grid cells). All 5887 site-level direct measurements were aggregated into 956 grid cells. Different colors indicate the magnitude of soil $\delta^{15}N$ (Unit: ‰).

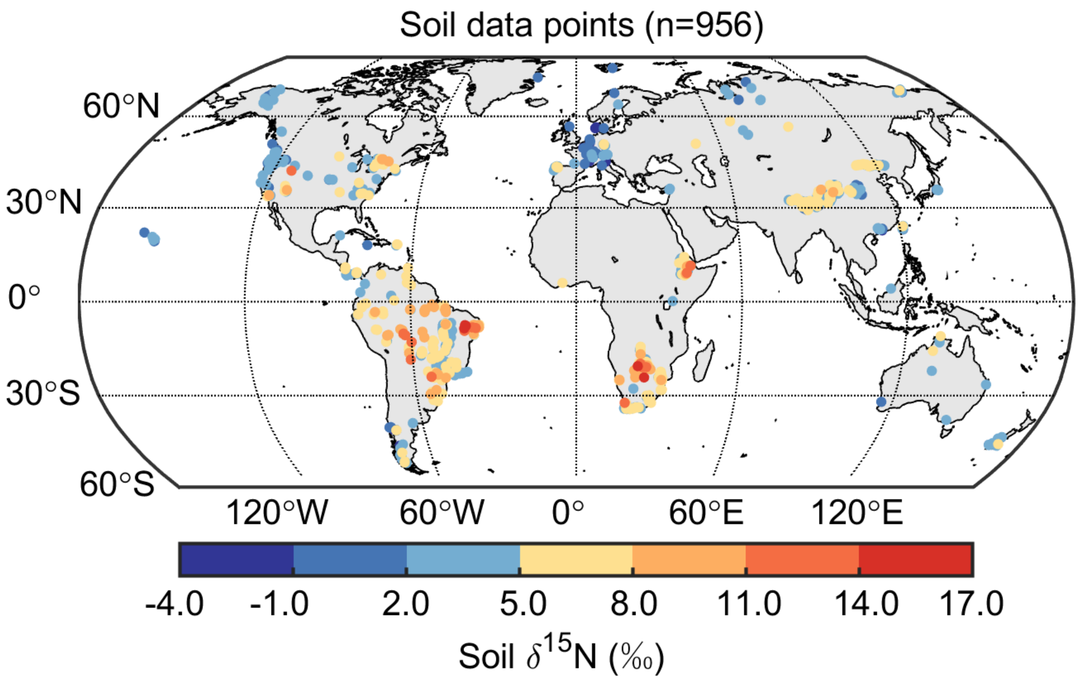

Supplementary Figure 2. Training and testing of the Random Forest (RF) model. The correlation between observed and predicted soil $\delta^{15}N$ for the training (a) and Out of Bag (OOB) samples (b).

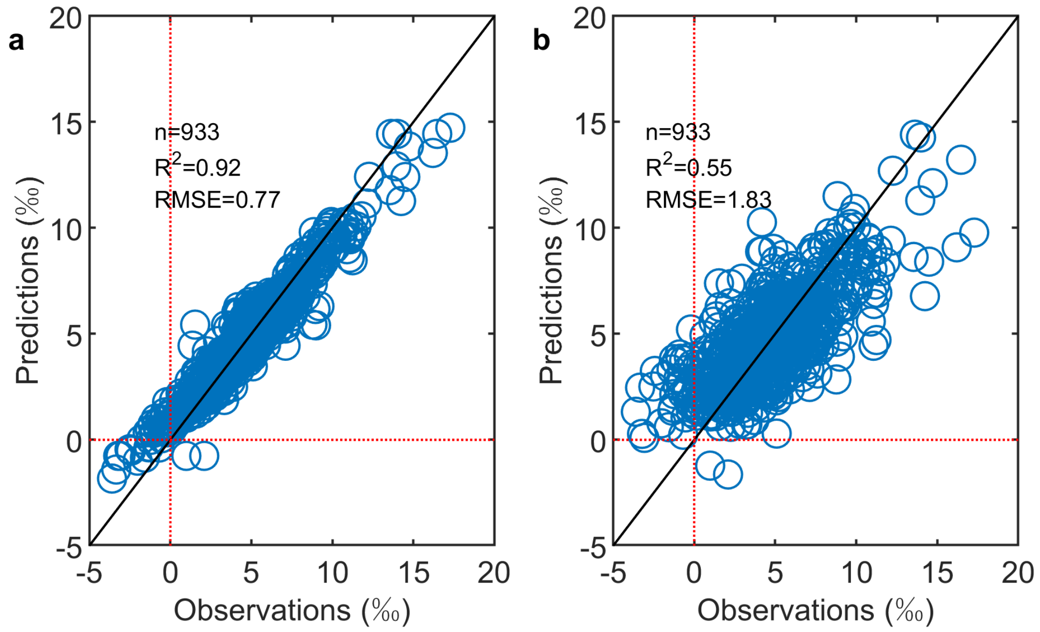

Supplementary Figure 3. Frequency distributions of the explained variance for the *K*-fold (*K*=10) cross validation. (a) Training samples. (b) Testing samples. The explained variance was represented by the coefficient of determination. The red vertical lines indicate the means of explained variance for the training and testing.

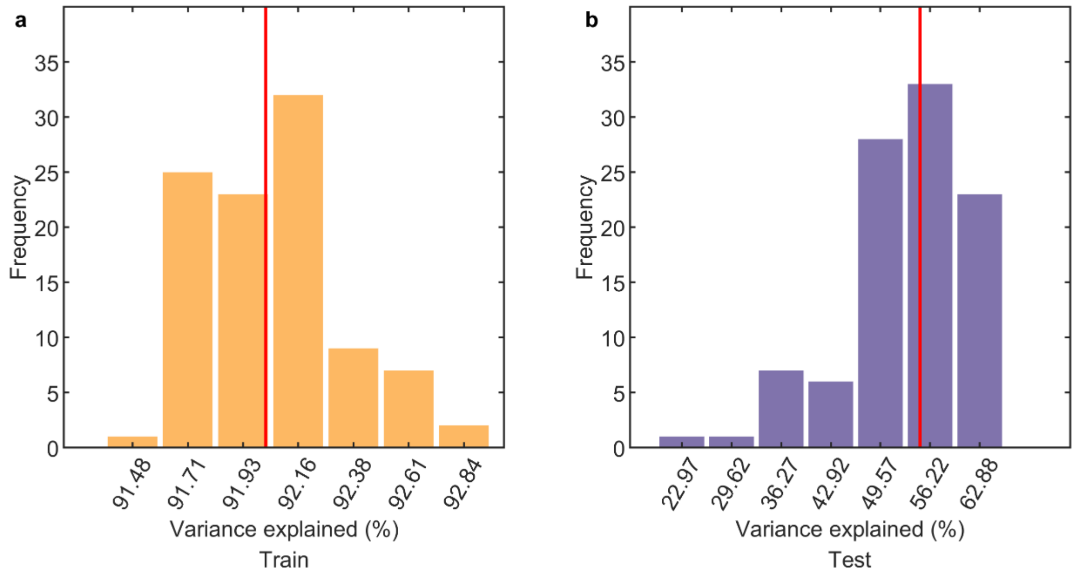

Supplementary Figure 4. Global maps of soil $\delta^{15}N$ and its standard deviation (SD). (a) and (b) are produced by the Random Forest (RF) model with N depositions (in the form of NH_x and NO_y; two of the 16 predictors) from Tian *et al.*[3]; (c) and (d) are produced by the RF model with the N depositions from European Monitoring and Evaluation Programme (EMEP)[4]. All crop and pastural areas are excluded from the analysis and represented by grey regions in the plots.

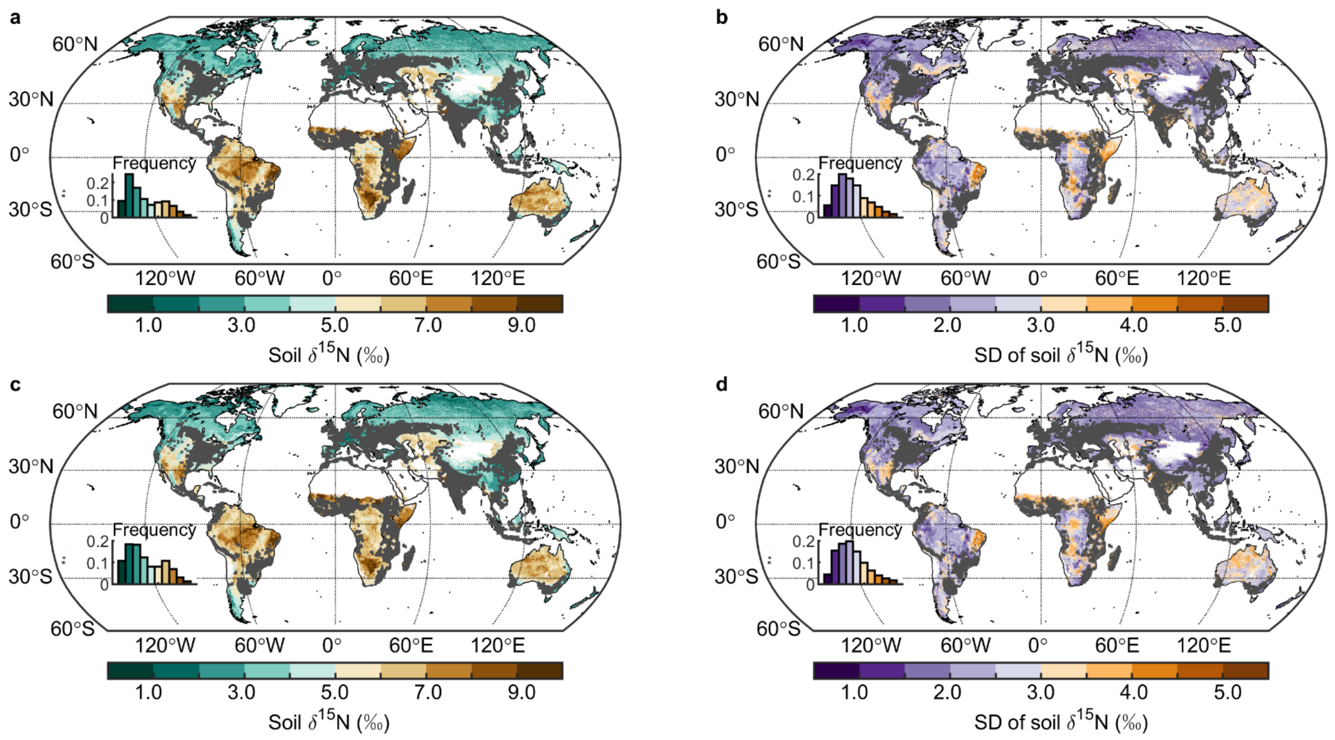

Supplementary Figure 5. Comparison of the validities of the linear regression model in Amundson *et al.*[6] and the Random Forest (RF) model in this study. The correlation between the observed and predicted soil $\delta^{15}N$ for the linear regression model (a) and the RF model (b).

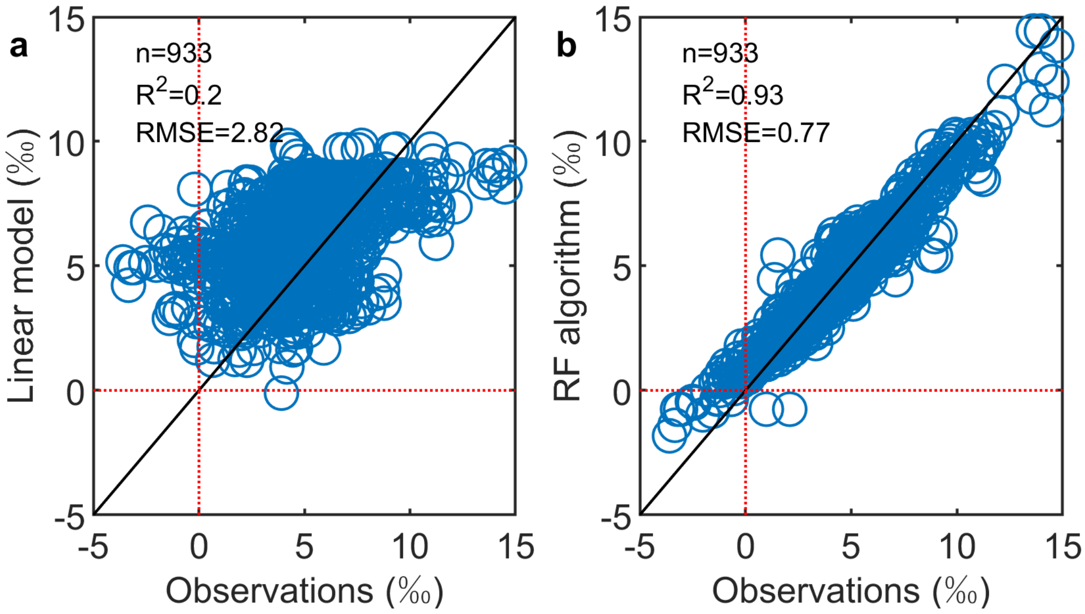

Supplementary Figure 6. Global maps of isotope-benchmarking based f_{denit} derived using different N inputs. All six maps of isotope-based f_{denit} share the same rock N flux from Houlton *et al.*[7]. N deposition for the maps in the left column (a, c and e) was from Tian *et al.*[3]; for the right column (b, d and f), it was from European Monitoring and Evaluation Programme (EMEP)[4]. Biological nitrogen fixation (BNF) was simulated using the Carnegie–Ames–Stanford Approach for Carbon, Nitrogen and Phosphorus cycles (CSCA-CNP) model, with the maps in the first (a and b), second (c and d), and third (e and f) rows corresponding to the use of Method A, Method B, and Method C, respectively, from Peng *et al.*[8]. All crop and pastural areas were excluded from the analysis and are indicated by the grey regions.

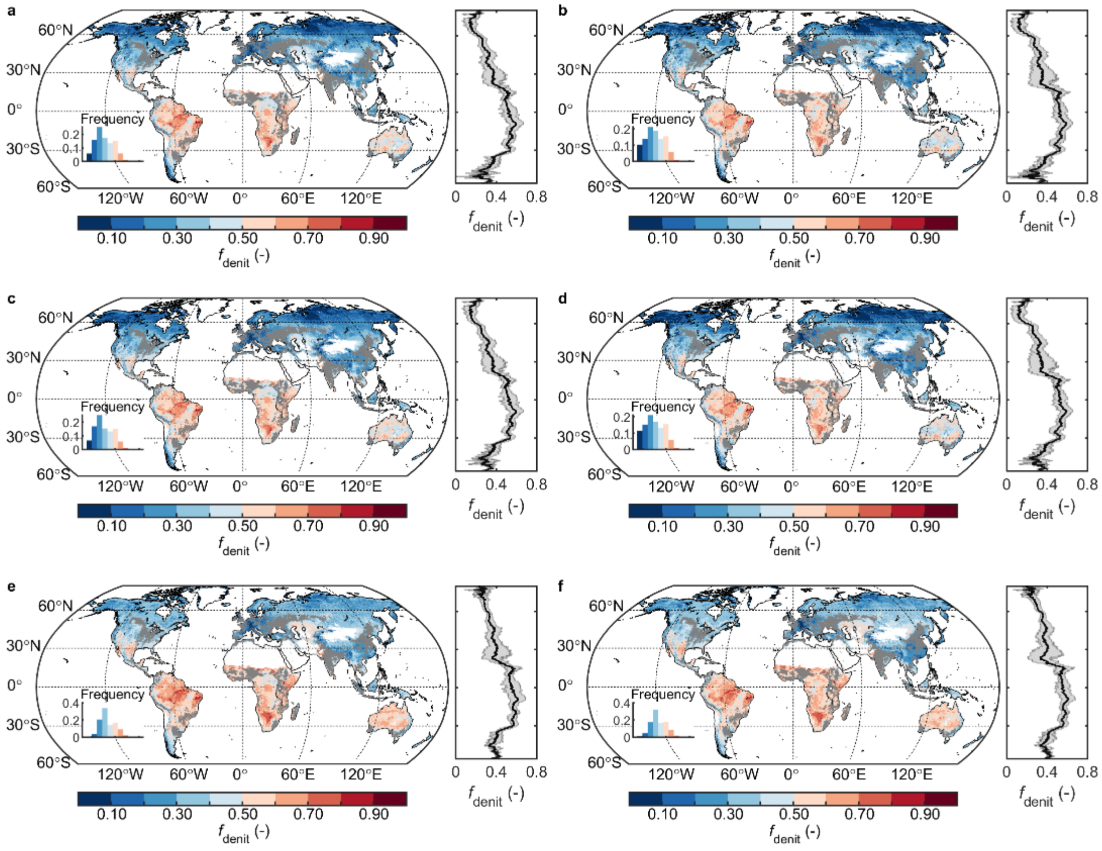

Supplementary Figure 7. Comparison of the global map of isotope-benchmarking based f_{denit} with previous estimates. (a) The global map of isotope-benchmarking f_{denit} derived in this study. (b) The global map of isotope-based f_{denit} derived from the global soil $\delta^{15}N$ map from Amundson *et al.*[6]. (c) The global map of land surface model based f_{denit} obtained from Goll *et al.*[9]. In (b), the parameterization of isotope mass balance equations is the same as that in Houlton *et al.*[5]. In (c), the global map of f_{denit} was derived by first obtaining a $\delta^{15}N$-related parameter by regression analysis, and then estimating the global pattern of f_{denit} by using global maps of the indicators of temperature and leaching simulated by JSBACH (Jena Scheme for Biosphere–Atmosphere Coupling in Hamburg) model[9].

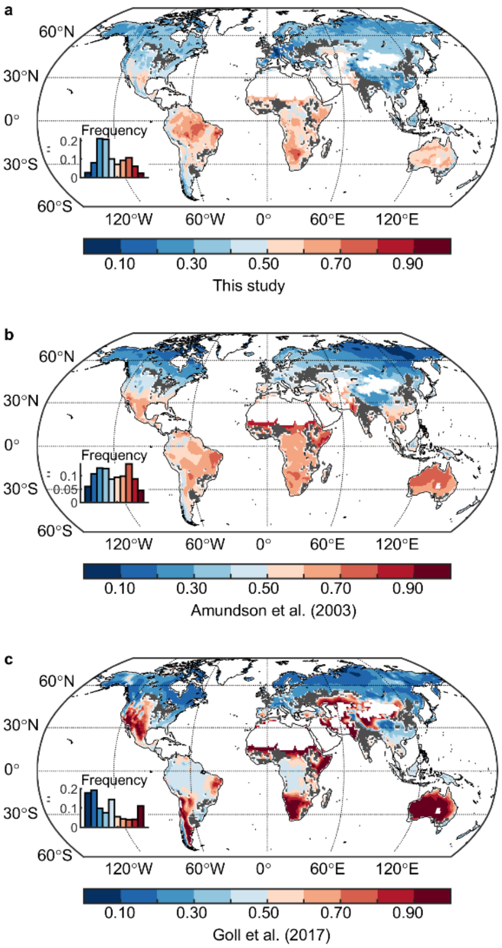

Supplementary Figure 8. Global maps of the standard deviations (SDs) of the isotope-benchmarking based f_{denit}. (a) shows the mean of six ensembles of global SD maps for f_{denit}. (b) shows the SD across six ensembles of global maps of f_{denit}.

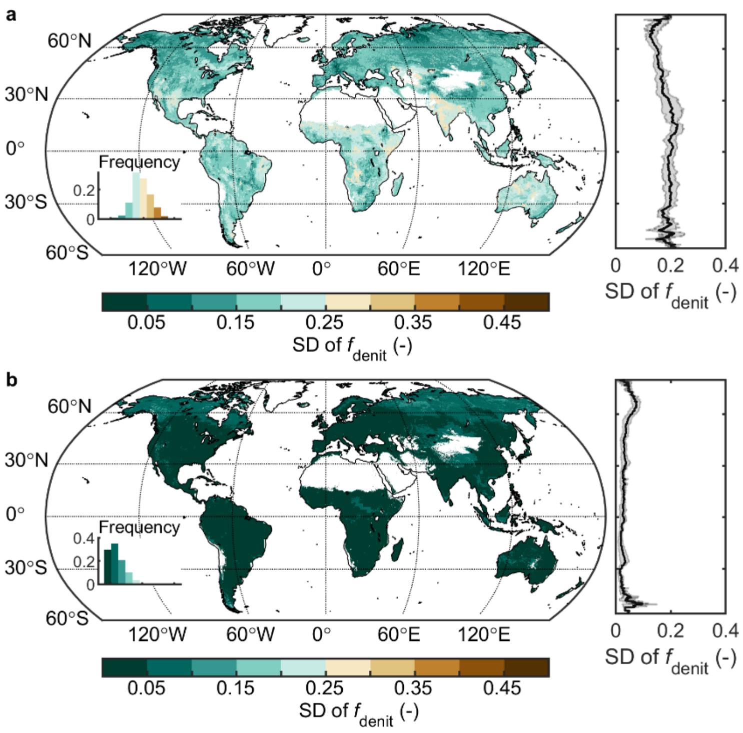

Supplementary Figure 9. Comparison of latitudinal gradients of f_{denit} simulated by the CMIP6 Earth System Models (ESMs) with that of the isotope-benchmarking based f_{denit} from this study.

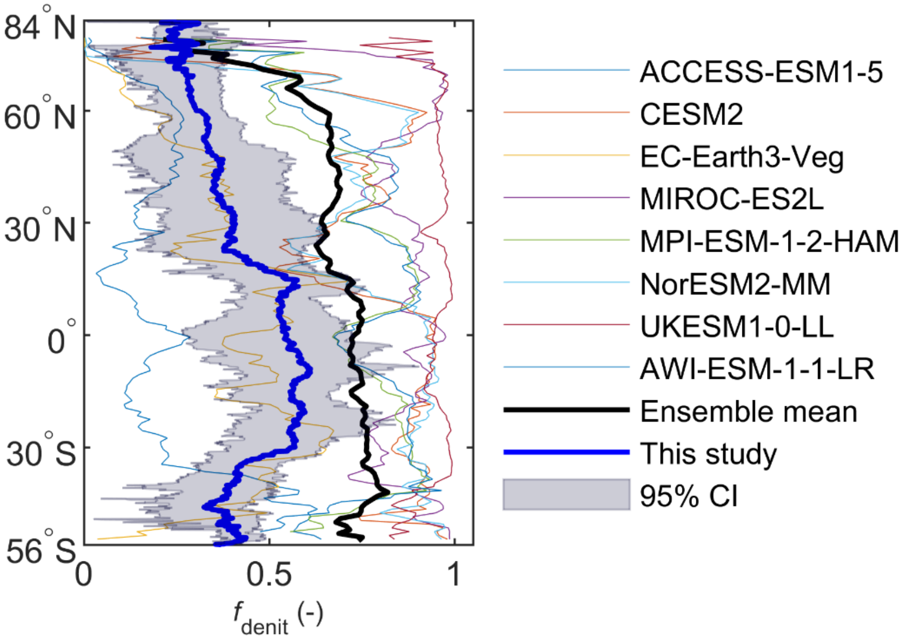

Supplementary Figure 10. The sensitivity of the global map of isotope-based f_{denit} to the isotope effect of denitrification ($\varepsilon_{\text{denit}}$). (a), (b), (c), and (d) are global maps of isotope-based f_{denit} derived from global constant $\varepsilon_{\text{denit}}$ at 10‰, 13‰, 16‰, 20‰, respectively. (e) and (f) are global maps of isotope-based f_{denit} derived from two temperature-dependent formulations of $\varepsilon_{\text{denit}}$: (e) $\varepsilon_{denit} = g_1(T) = a + b/T$, and (f) $\varepsilon_{denit} = g_2(T) = a + b/(T_{\max} + T_{\min} - T)$. The parameters a and b were estimated by setting the maximum and minimum values of ε_{denit} to 20‰ and 10‰, respectively. All crop and pastural areas were excluded from the analysis and are represented by grey regions in the plots.

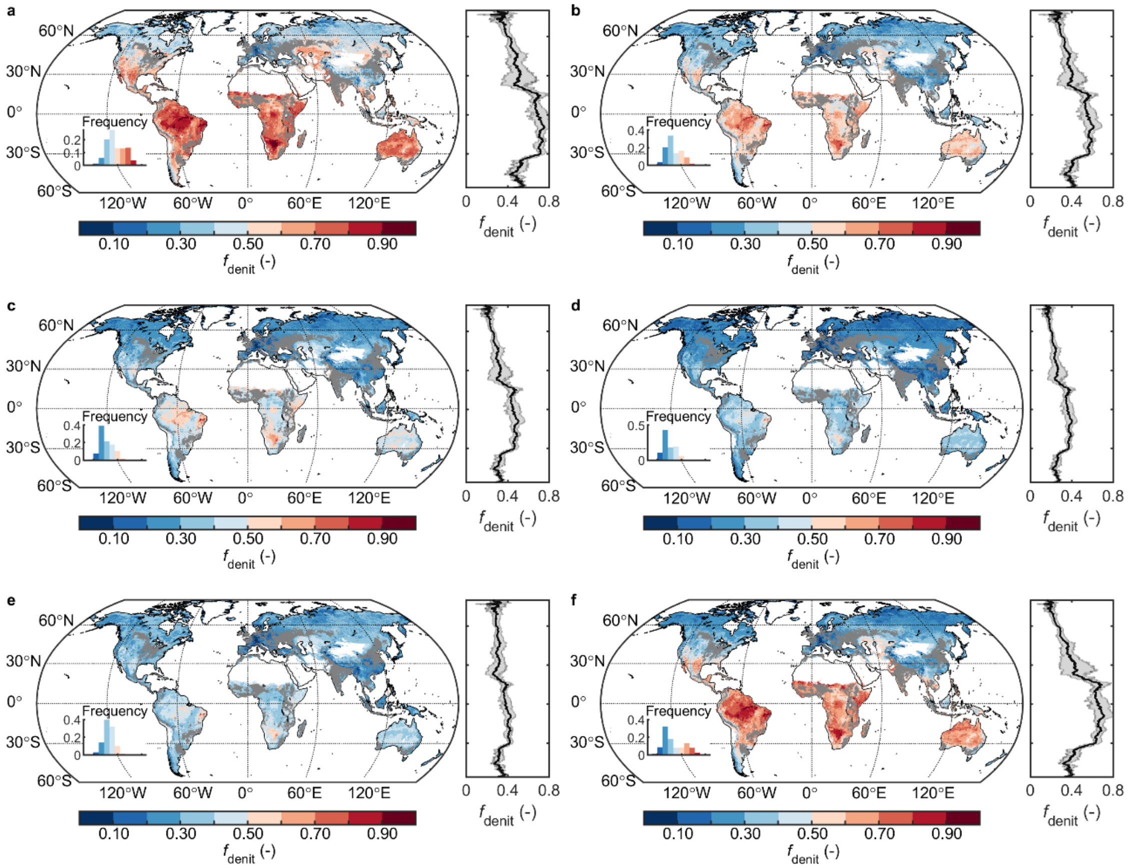

Supplementary Figure 11. The linear relationship between the fraction of grid cells with f_{denit}>0.8 and f_{denit} for the 13 Earth System Models (ESMs) in CMIP6. The black line and shaded area are the best-fit regression line and 95% confidence interval (CI), respectively.

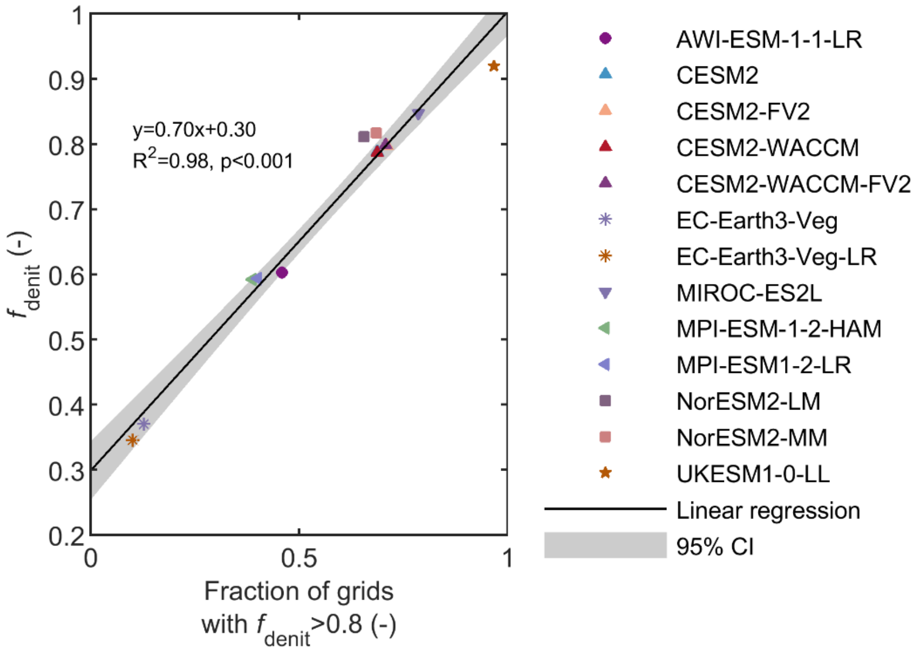

Supplementary Figure 12. The seasonal and interannual variations of f_{denit} simulated by the CMIP6 Earth System Models (ESMs). (a) and (b) show the seasonal and interannual variations, respectively, of the global mean of f_{denit}, during the period 1850–2014. (c) is the negative correlation between the mean and SD of f_{denit} across the ESMs. The black line and shaded area are the best-fit regression line and 95% confidence interval (CI), respectively. This negative correlation supports the hypothesis that an ESM with a higher f_{denit} is more likely to be constrained by the upper bound (i.e., 1) and corresponds to a lower variation. In panel (c), the ACCESS-ESM1-5 (unfilled symbol) was excluded from the linear regression since it is far away from the confidence interval.

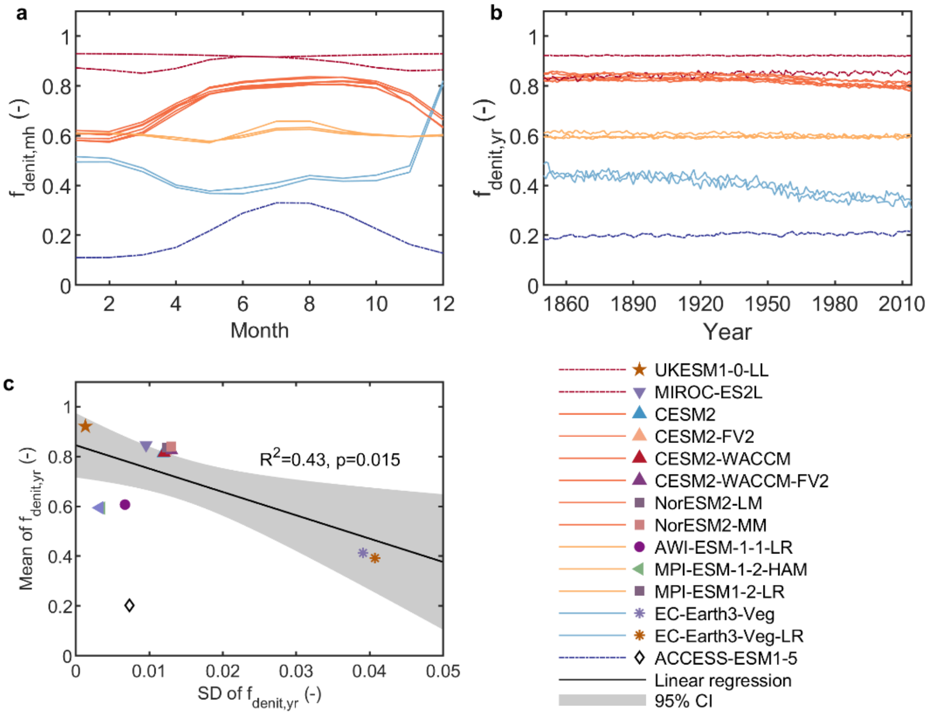

Supplementary Figure 13. Comparison of the global pattern of f_{denit} in summer as simulated by the CMIP6 Earth System Models (ESMs) with the isotope-benchmarking based estimate in this study. (a) Global map of the isotope-benchmarking based f_{denit} of this study. (b)-(i) Global maps of f_{denit} in summer during the period 2005–2014 simulated by CMIP6 ESMs (name of ESM on the top of the map), with each representing a family of ESMs with similar patterns of f_{denit}. In each panel, the histogram in the bottom left corner shows the frequency distribution of f_{denit} values across the globe. All crop and pastural areas were excluded from the analysis and are represented by the grey regions in the plots.

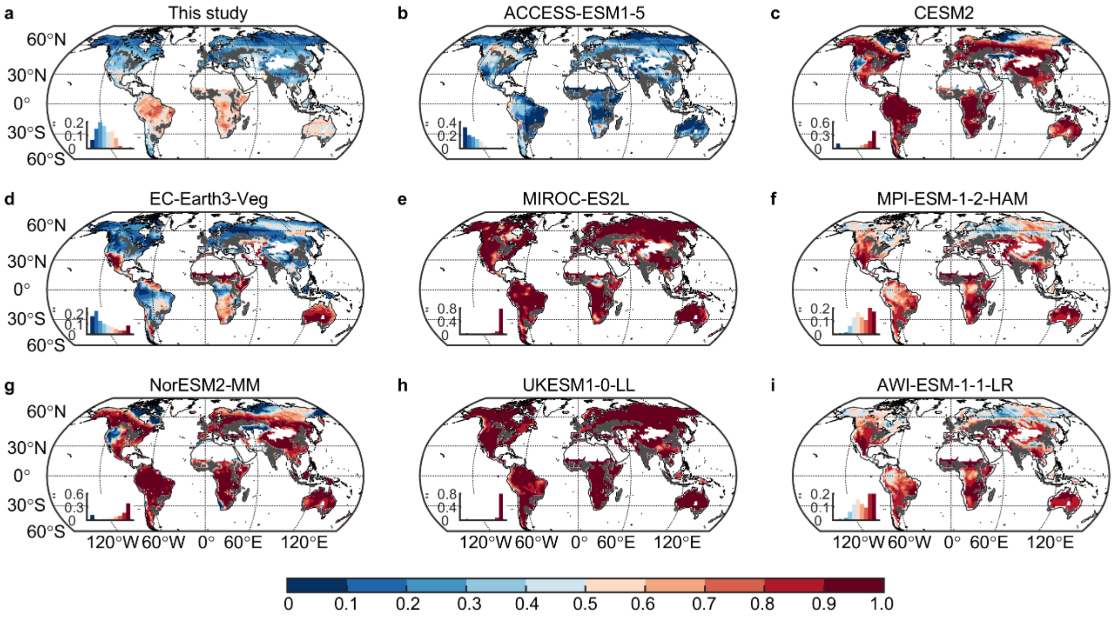

Supplementary Figure 14. Comparison of the global pattern of f_{denit} in winter as simulated by the CMIP6 Earth System Models (ESMs) with the isotope-benchmarking based estimate in this study. (a) Global map of the isotope-benchmarking based f_{denit} of this study. (b)-(i) Global maps of f_{denit} in winter during the period 2005–2014 simulated by CMIP6 ESMs (name of ESM on the top of the map), with each representing a family of ESMs with similar patterns of $f_{denit.}$ In each panel, the histogram in the bottom left corner shows the frequency distribution of f_{denit} values across the globe. All crop and pastural areas were excluded from the analysis and are represented by the grey regions in the plots.

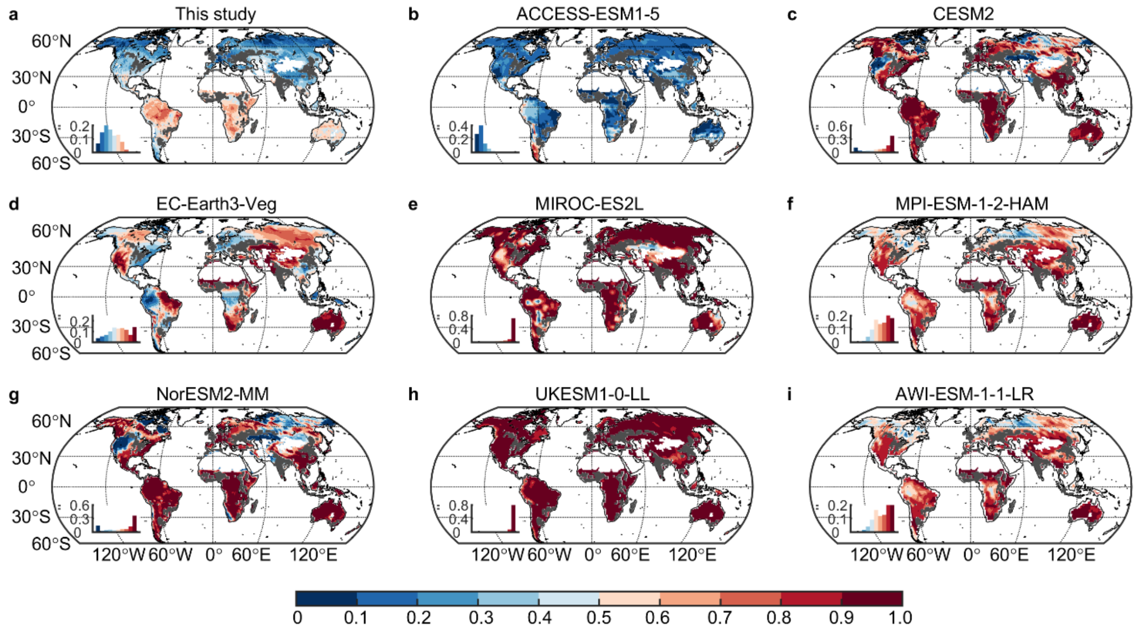

Supplementary Figure 15. Global maps of denitrification N loss estimated with isotope-benchmarking based f_{denit} under steady state conditions (total N losses equal to total N inputs). The derivations of all the maps of denitrification N loss share the same rock N flux from Houlton *et al.*[5]. N deposition for the maps in the lefthand column (a, c and e) was from Tian *et al.*[3]; for the righthand column (b, d, and f), it was from European Monitoring and Evaluation Programme (EMEP)[4]. Biological nitrogen fixation (BNF) was simulated using the Carnegie–Ames–Stanford Approach for Carbon, Nitrogen and Phosphorus cycles (CSCA-CNP) model, with the maps in the first (a and b), second (c and d), and third (e and f) rows corresponding to the use of Method A, Method B, and Method C, respectively, from Peng *et al.*[8]. In each panel, the lines and shaded area on the right of the map are the latitudinal mean and 95% confidence interval, respectively. All crop and pastural areas were excluded from the analysis and are represented by grey regions.

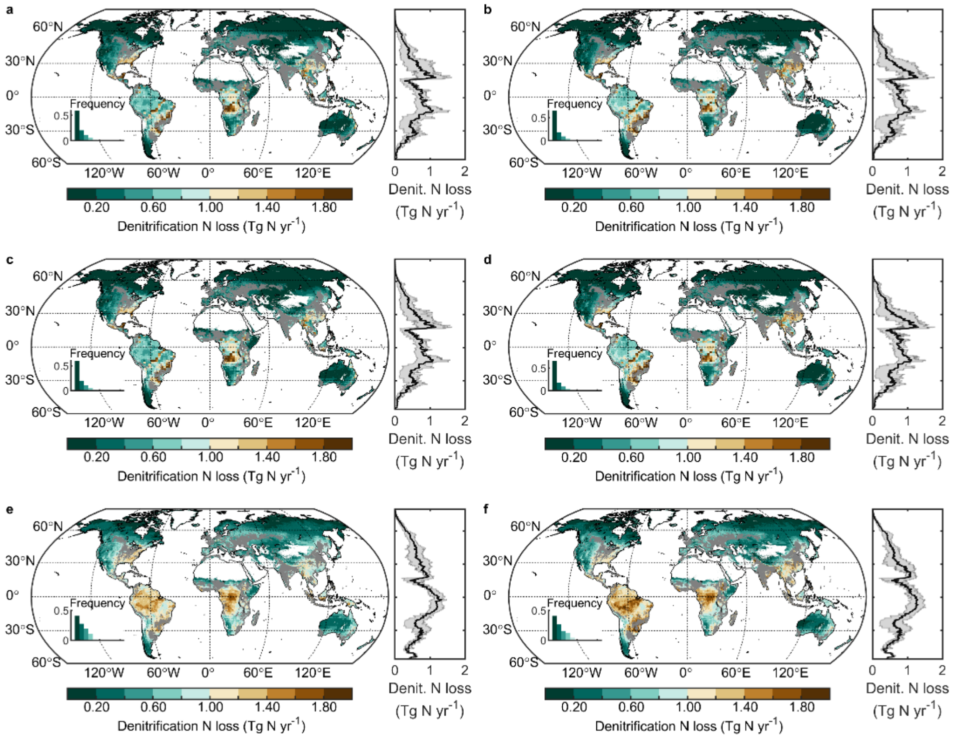

Supplementary Figure 16. The effects of the terrestrial N sink on soil $\delta^{15}N$ and isotope-based f_{denit}. The N fluxes and their isotope signals for an ecosystem (a) under steady state, (b) with a terrestrial N sink of 25 Tg N yr^{-1} and (c) with a terrestrial N sink of 44 Tg N yr^{-1}. In (b) and (c), the effects of the terrestrial N sink were assessed by simulating the box-model with a Eulerian method for 50 years. In all cases, the isotope-based f_{denit} were derived using Eq. (2) in Methods, revealing the effects of the N sink on isotope-based f_{denit}.

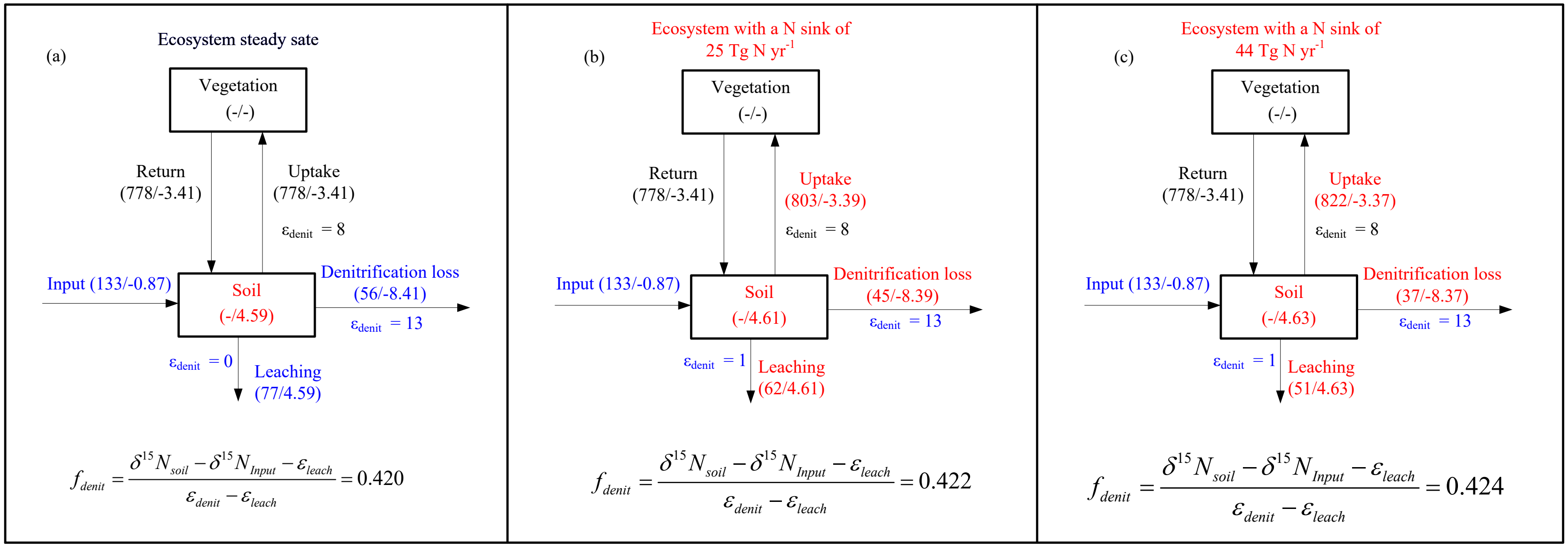

Supplementary Figure 17. Predictor importance as identified by the Random Forest (RF) algorithm. The predictors in an order of descending importance are abundance of N fixing bacteria (Nfix), Temperature (T), abundance of Ecto-mycorrhizal fungi (ECM), Aridity index–Precipitation over potential evapotranspiration (P/PET), N deposition in the form of NO_y (NO_y), abundance of Arbuscular mycorrhizal fungi (AM), fraction of silt (Silt), N deposition in the form of NH_x (NH_x), Organic Carbon (OC), Carbon to nitrogen ratio (C/N), Precipitation (P), Bulk density (BD), soil acidity or alkalinity (pH), fraction of caly (Clay), Gross primary production (GPP), and fraction of sand (Sand).

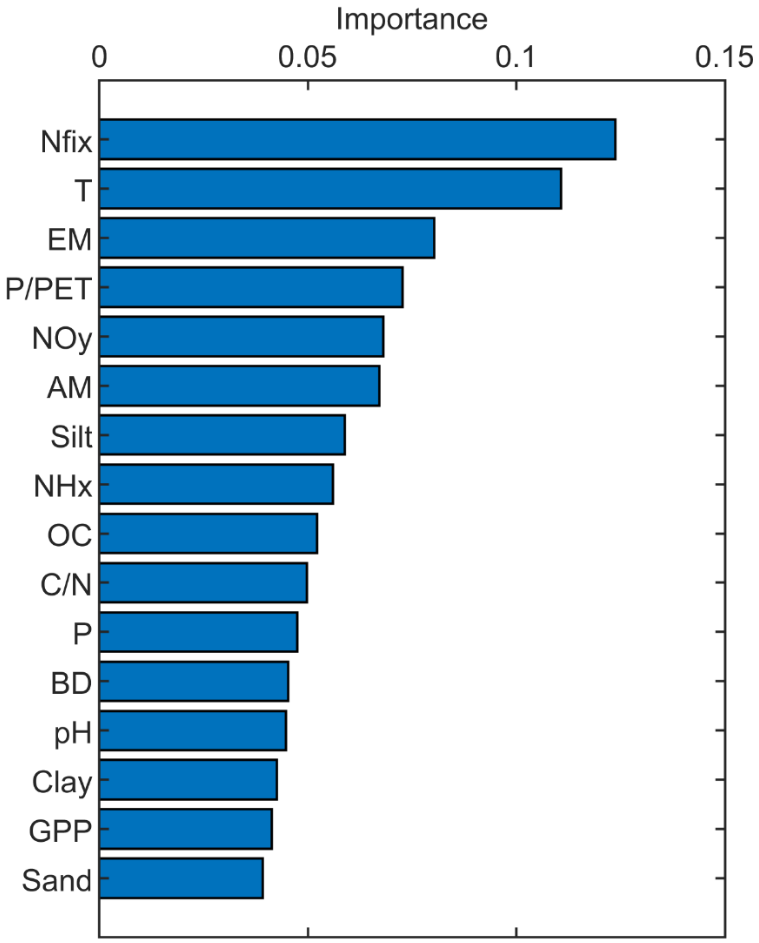

Supplementary Figure 18. The linear regression relationship between soil $\delta^{15}N$ observations and six leading predictors. These leading predictors are identified by random forest model: (a) N fixing bacteria (Nfixer), (b) Temperature (T), (c) Ectomycorrhizal fungi (ECM), (d) aridity index–precipitation over potential evapotranspiration (P/PET), (e) N deposition in form of NOy, (f) Arbuscular mycorrhizal fungi (AM).

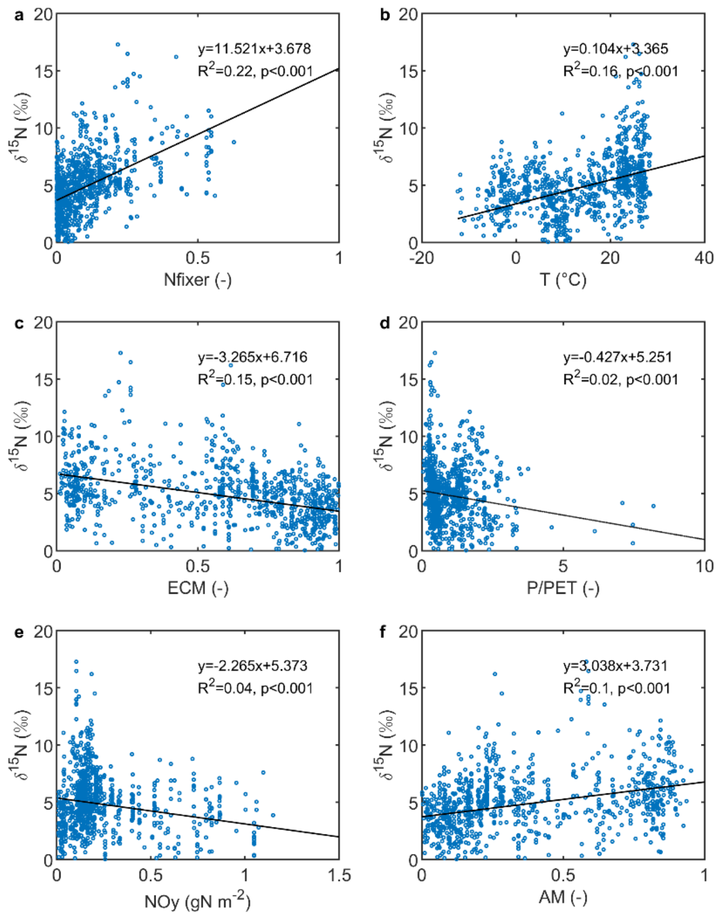

Supplementary Figure 19. The partial dependence plot between the Random Forest (RF)-predicted $\delta^{15}N$ and six leading predictors. Relationships between RF-predicted $\delta^{15}N$ and (a) N fixing bacteria (Nfixer), (b) Temperature (T), (c) Ectomycorrhizal fungi (ECM), (d) aridity index–precipitation over potential evapotranspiration (P/PET), (e) N deposition in form of NOy, (f) Arbscular mycorrhizal fungi (AM). The partial dependence was quantified by a well-established python package, parital_dependence, and plotted by the Matlab software.

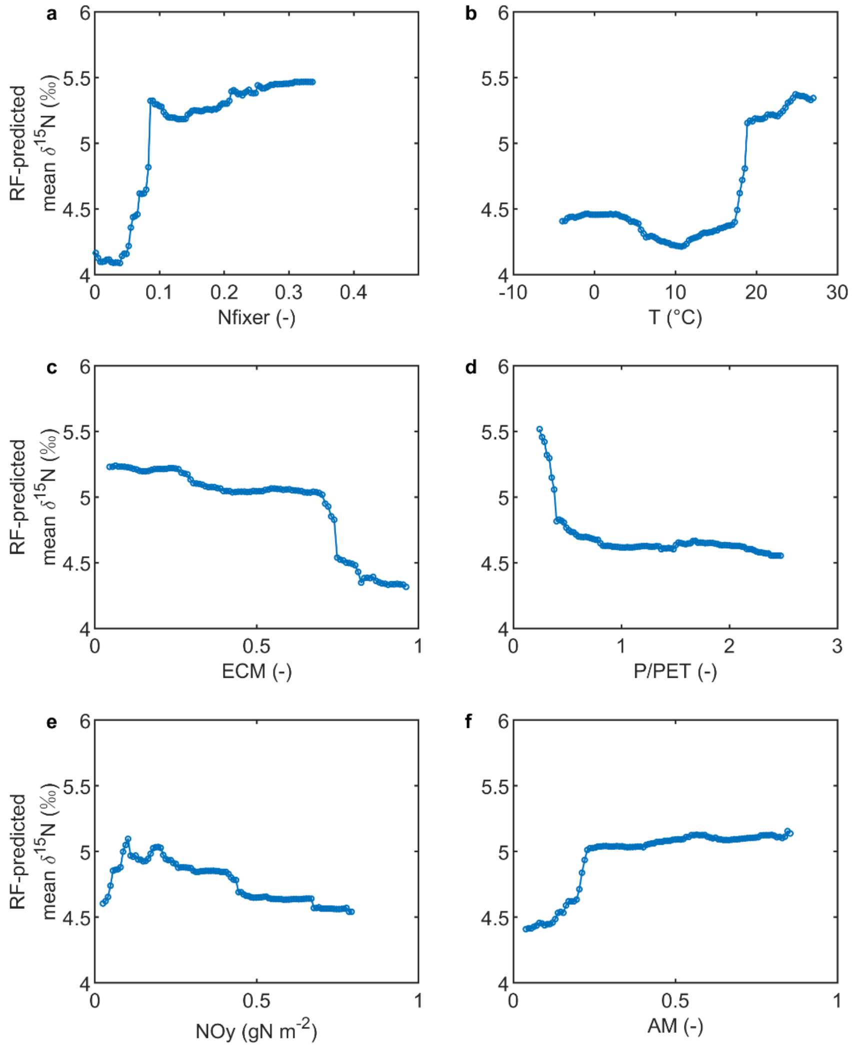

Supplementary Figure 20. Comparison of the linear regression relationships between soil δ^{15}N observations and mean annual temperature (MAT) and precipitation (MAP) in this study with those in Craine *et al.* (2015). (a) and (b) are linear regression relationship between soil δ^{15}N observations and MAT and MAP, respectively, in this study. (c) and (d) are linear regression relationship between soil δ^{15}N observations and MAT and MAP, respectively, in Craine *et al.* (2015).

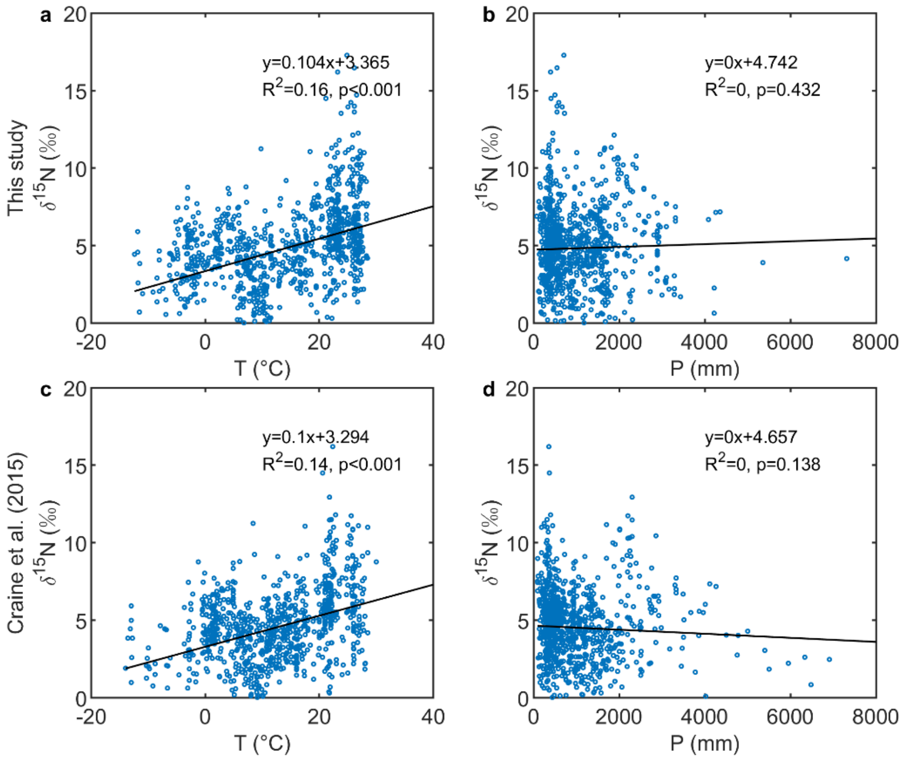

Supplementary Figure 21. Comparison of the nonlinear relationships between soil δ^{15}N observations and mean annual temperature (MAT) and precipitation (MAP) in this study with those in Craine *et al.* (2015). (a) and (c) are piecewise linear relationships between soil δ^{15}N observations and MAT (with a threshold of 8.5 °C), in this study and Craine *et al.* (2015), respectively. (b) and (d) are regression relationships between soil δ^{15}N observations and log MAP, in this study and Craine *et al.* (2015), respectively.

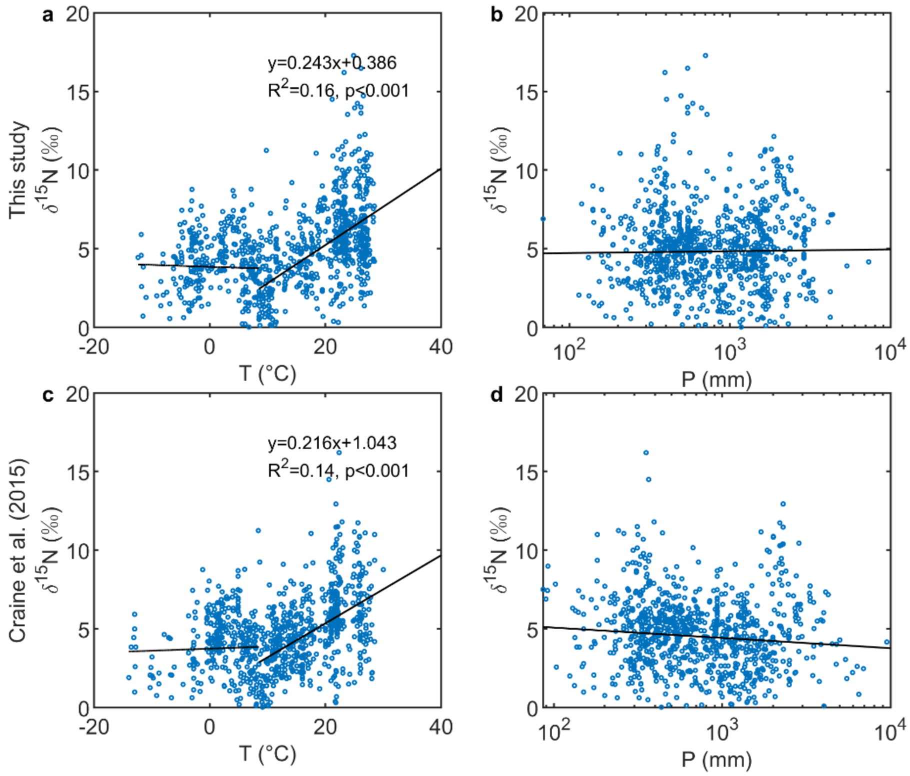

Supplementary Figure 22. Global maps of rock $\delta^{15}N$ signals. (a) is a medium level of rock $\delta^{15}N$; (b) and (c) are the lower and upper bounds of the rock $\delta^{15}N$, respectively. The global rock $\delta^{15}N$ maps were produced based on the surficial lithologic composition of Earth's continental surfaces generated by Dürr *et al.*[26] and $\delta^{15}N$ signals of different rock types summarized in Supplementary Table 10.

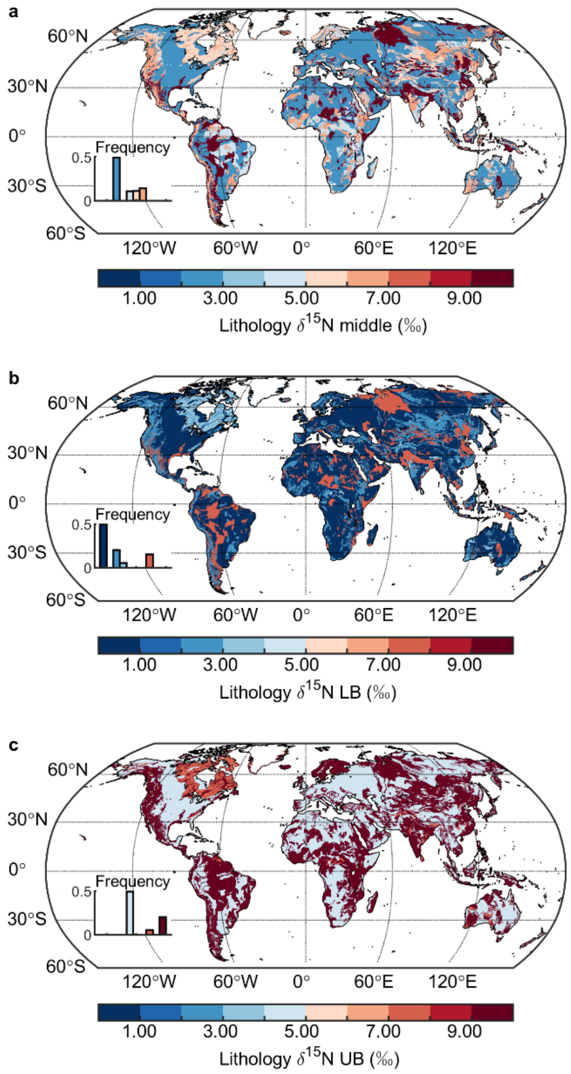

Supplementary Figure 23. The sensitivity of global maps of isotope-based f_{denit} to the standard deviations of input $\delta^{15}N$ (SD_{input}). The SD_{input} of input $\delta^{15}N$ was set at different levels of mean input $\delta^{15}N$ ($Mean_{input}$): (a) SD_{input} = 3% $Mean_{input}$, (b) SD_{input} = 4% $Mean_{input}$, (c) SD_{input} = 6% $Mean_{input}$, and (d) SD_{input} = 7% $Mean_{input}$. All crop and pastural areas were excluded from the analysis and are represented as grey regions in the plots.

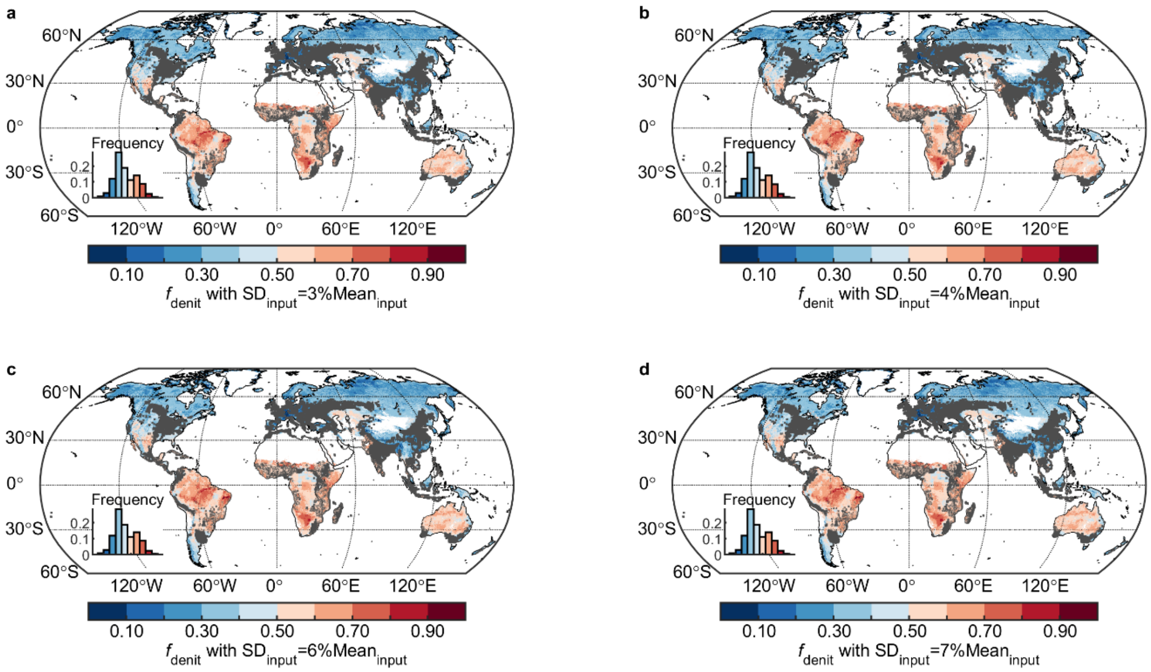

Supplementary Tables

Supplementary Table 1. The isotope-benchmarking based fraction of denitrification (f_{denit}) and denitrification N loss under steady state conditions, i.e., denitrification N loss = $f_{\text{denit}}\times$Ninput.

	N deposition input	BNF input	Area (M km^2)	Rock N (Tg yr^{-1})	N deposition (Tg yr^{-1})	BNF (Tg yr^{-1})	Total Input (Tg yr^{-1})	δ_{input} (‰)	δ_{soil} (‰)	f_{denit}	Gas Loss	Gas (LB of 95%CI)	Gas (UB of 95% CI)
1	EMEP[4]	CSCA-A[8]	84.33	9.78	41.86	45.91	97.55	-0.53	4.83	0.41	40	37	44
2	EMEP[4]	CSCA-B[8]	84.33	9.78	41.86	43.95	95.59	-0.50	4.87	0.41	39	37	43
3	EMEP[4]	CSCA-C[8]	84.33	9.78	41.86	81.22	132.85	-0.92	4.80	0.43	57	55	63
4	Tian et al.[3]	CSCA-A[8]	84.33	9.78	38.68	45.91	94.37	-0.55	4.81	0.41	38	36	43
5	Tian et al.[3]	CSCA-B[8]	84.33	9.78	38.68	43.95	92.41	-0.52	4.84	0.41	38	35	42
6	Tian et al.[3]	CSCA-C[8]	84.33	9.78	38.68	81.22	129.68	-0.94	4.76	0.43	56	53	62
	Mean		84.33	9.78	40.27	57.03	107.08	-0.66	4.82	0.42	45	42	50
	SD		0	0	1.74	18.76	18.84	0.21	0.04	0.01	9	9	10

Supplementary Table 2. The sensitivities of the isotope-based fraction of denitrification (f_{denit}) and denitrification N loss to the isotope effect of denitrification (ε_{denit}), under three levels (medium, low and high) of rock δ^{15}N. Note that g_1(T) and g_2(T) are two temperature (T)-dependent scenarios with ε_{denit} inferred from gridded surface air temperature: $g_1(T) = a + b/T$, and $g_2(T) = a + b/(T_{max} + T_{min} - T)$.

	ε_{denit}	δ_{input}	f_{denit}	LB of f_{denit}	UB of f_{denit}	Denit. N Loss	LB of denit. N loss	UB of denit. N loss
Rock ^{15}N at medium level	10	-0.94	0.55	0.51	0.59	73	67	78
	13	-0.94	0.43	0.40	0.47	57	53	62
	16	-0.94	0.35	0.32	0.38	46	43	51
	20	-0.94	0.28	0.26	0.31	37	34	40
	g_1(T)	-0.94	0.47	0.43	0.51	62	57	67
	g_2(T)	-0.94	0.33	0.30	0.36	43	40	47
Rock ^{15}N at lower bound	10	-1.14	0.57	0.53	0.61	75	70	80
	13	-1.14	0.45	0.41	0.48	59	54	64
	16	-1.14	0.36	0.34	0.39	48	44	52
	20	-1.14	0.29	0.27	0.32	38	35	42
	g_1(T)	-1.14	0.48	0.45	0.52	64	59	69
	g_2(T)	-1.14	0.34	0.31	0.37	45	42	49
Rock ^{15}N at upper bound	10	-0.75	0.53	0.49	0.57	70	65	76
	13	-0.75	0.42	0.39	0.45	55	51	60
	16	-0.75	0.34	0.31	0.37	45	41	49
	20	-0.75	0.27	0.25	0.30	36	33	39
	g_1(T)	-0.75	0.46	0.42	0.49	60	55	65
	g_2(T)	-0.75	0.32	0.29	0.35	42	39	46

Supplementary Table 3. Summary of the representations of denitrification and leaching N losses in the CMIP6 Earth System Models (ESMs).

C-N module	ESMs	Gaseous N loss	Leaching N loss	References
CASA-CNP	ACCESS-ESM1-5	$F_{n,gas} = f_{ngas}\max(0, F_{n,net})$ where f_{ngas} equals 0.05 year^{-1}, and $F_{n,net}$ is the net mineralization flux.	$F_{n,leach} = f_{nleach}N_{min}$ where f_{nleach} equals 0.5 yr^{-1}, and N_{min} is the mineral N.	Wang *et al.*[27]
CLM5/CLM4.5	CESM2 family; NorESM2 family; AWI-ESM-1-1-LR	$f_{nitr,p} = [NH_4^+]\ k_{nitr} f(T)\ f(H_2O)\ f(pH)$ $f_{denit,p} = \min\{f(\text{decomp}), f([NO_3^-])\}frac_{anox}$ where $f_{nitr,p}$ and $f_{denitr,p}$ are rates of potential nitrification and denitrification, respectively; $[NH_4^+]$ is the concentration of NH_4^+, and k_{nitr} is the nitrification rate (10 % day^{-1}) ; $f(T)$, $f(H_2O)$, and $f(pH)$ are impacts of soil temperature, soil moisture and pH on the nitrification; $f(\text{decomp})$, and $f([NO_3^-])$ are carbon- and nitrate-limited denitrification rate functions, respectively;	$NF_{leached}=(NS_{sminn}\ sf/WS_{tot\_soil})\ Q_{dis}$ where sf is a constant fraction of soluble inorganic N, NS_{sminn} is the soil mineral N, WS_{tot_soil} is total mass of soil water content integrated over the column; Q_{dis} is the rate of hydrologic discharge from the soil column to river	Technical note of CLM5.0/CLM4.5

		$frac_{anox}$ is the fraction of anoxic microsites in the soil.<br>N_2O emission is assumed as fractions of nitrification and denitrification fluxes.		
LPJ-GUESS v4	EC-Earth3 family	$F_{denit} = 0.01 k_{max} f(T_{soil})\, f(W)\, f(S)$<br>$f(T_{soil}) = 0.0326+0.00351 T_{soil}^{1.652}-(T_{soil}/41.748)^{7.19}$<br>$f(W)=\begin{cases} 0.00037\sigma^2-0.0748\sigma+4.13, & \sigma\geqslant 60 \\ \exp\left(-\frac{(\sigma-60)^2}{800}\right), & \sigma<60 \end{cases}$<br>$f(S)=1-0.75S$<br>where k_{max} is a prescribed maximum rate, T_{soil} is the soil temperature, W is the soil moisture, and S is soil fractional content of silt and clay. $\sigma=100\theta/\theta_{max}$ is a proxy for percentage of water-filled pore spaces in the soil, θ is current soil water content, and θ_{max} is soil water saturation capacity as a proportion of soil column depth.	N loss from leaching was computed daily as the sum of leached soluble organic N and leached mineral N	Smith *et al.*[28]
JSBACH v3.20	MPI-ESM family	$F_{denit}=\alpha k_{denit} N_{smin}$	$F_{leach} = f_s N_{smin} f_{h2o}$<br>where f_s is the fraction of soluble mineral N, and f_{h2o}	Goll *et al.*[9]

		where k_{denit} =0.002 day^{-1} is the maximum rate of denitrification, α is an indicator for moisture stress, and N_{smin} is the soil mineral pool	is the fraction of soil water lost to rivers	
VISIT-e	MIROC-ES2L	$F_{denitr} = \beta_{WFPS\text{-}den}\min(F_{den}(N_{NO3}), F_{den}(R_H))$<br>$F_{N2O\text{-}nit} = \beta_{WFPS\text{-}nit}\beta_{pH}\beta_t\,(K_{max}+F_{max}NH_4^+)$<br>where $\beta_{WFPS\text{-}den}$ is the impact of water filled pores in soil (WFPS) on denitrification rate, $F_{den}(N_{NO3})$ and $F_{den}(R_H)$ are maximum total gas fluxes for a given soil NO_3 concentration and a given heterotrophic respiration rate, respectively.<br>$\beta_{WFPS\text{-}nit}$, β_{pH}, and β_t are the effects of soil moisture or WFPS, soil pH, and soil temperature, respectively, on nitrification; K_{max} is the soil-specific N turnover coefficient; F_{max} is the maximum nitrification gas flux; and NH_4^+ is the effect of soil NH_4^+ concentration on nitrification.	Not found	Inatomi *et al.*[29]
JULES-ES-1.0	UKESM1-0-LL	$N_{gas} = f_{gas}\,(M_{tot} - I_{tot})$<br>NgasI = $\gamma_n N_{in}$ with γ_n =0.0028 day^{-1}	$N_{leach} = \alpha\,(N_{in}/\theta_{1m})\,Q_{subs}$<br>where θ_{1m} is the soil water content in the top 1 m of	Wiltshire *et al.*[30]

where f_{gas} is is a parameter that sets the fraction of the N flux that is emitted as gas to the atmosphere (0.01); M_{tot} and I_{tot} are the total fluxes of mineralization and immobilization, respectively. N_{gasI} is the additional gaseous N loss from inorganic N pool N_{in}	soil; Q_{subs} is the total subsurface runoff.

Supplementary Table 4. The biome-level estimates of total N inputs (Tg N yr^{-1}), f_{denit} (-) and denitrification N losses (Tg N yr^{-1}) under the steady state. The total N inputs were obtained with different combinations of N deposition (Tian *et al.*[3] and EMEP[4]) and biological nitrogen fixation (Method A, B, and C from Peng *et al.*[8]).

	TrBE	TrBR	TeNE	TeBE	TeBS	BrNE	BrBS	BrNS	C3 grass	C4 grass	Total
Area (M km^2)	12.6	7.5	4.4	4.4	4.8	6.8	5.4	3.7	23.4	11.4	84.3
Toal N Inputs (Tg N yr^{-1})											
**Tian+A**	18.0	9.9	7.3	7.0	8.6	4.4	4.4	1.7	19.0	14.1	94.4
**Tian+B**	18.1	9.9	7.2	6.8	8.4	4.1	4.2	1.7	17.9	14.1	92.4
**Tian+C**	25.3	12.2	8.4	8.6	9.9	8.2	7.3	3.8	28.0	18.0	129.7
**EMEP+A**	19.8	10.5	8.2	8.5	9.5	3.2	3.6	1.0	17.4	16.0	97.6
**EMEP+B**	19.2	10.3	8.1	8.4	9.4	3.2	3.5	1.0	16.7	15.8	95.6
**EMEP+C**	26.5	12.6	9.3	10.2	10.8	7.3	6.7	3.1	26.8	19.7	132.9
**Mean**	21.2	10.9	8.1	8.3	9.4	5.1	4.9	2.0	21.0	16.3	107.1
f_{denit} (-)											
**Tian+A**	0.51	0.53	0.37	0.32	0.37	0.26	0.29	0.23	0.33	0.48	0.41
**Tian+B**	0.51	0.53	0.37	0.32	0.37	0.25	0.28	0.23	0.33	0.48	0.41
**Tian+C**	0.55	0.55	0.39	0.34	0.38	0.32	0.34	0.31	0.37	0.50	0.43
**EMEP+A**	0.51	0.52	0.34	0.29	0.34	0.27	0.29	0.23	0.32	0.46	0.41
**EMEP+B**	0.52	0.53	0.35	0.30	0.35	0.26	0.29	0.22	0.33	0.46	0.41
**EMEP+C**	0.55	0.55	0.37	0.32	0.36	0.34	0.35	0.33	0.37	0.49	0.43
**Mean**	0.53	0.53	0.37	0.32	0.36	0.28	0.31	0.26	0.34	0.48	0.42
Denitrification N losses (Tg N yr^{-1})											
**Tian+A**	9.2	5.2	2.7	2.3	3.1	1.1	1.3	0.4	6.4	6.7	38.4
**Tian+B**	9.3	5.2	2.7	2.2	3.1	1.0	1.2	0.4	5.9	6.7	37.7
**Tian+C**	13.8	6.7	3.3	2.9	3.7	2.6	2.5	1.2	10.3	9.0	56.0
**EMEP+A**	10.0	5.4	2.9	2.5	3.3	0.9	1.1	0.2	6.0	7.3	39.7
**EMEP+B**	10.0	5.5	2.8	2.5	3.2	0.8	1.0	0.2	5.5	7.3	39.0
**EMEP+C**	14.6	6.9	3.5	3.2	3.9	2.5	2.3	1.0	10.0	9.6	57.4
**Mean**	11.2	5.8	3.0	2.6	3.4	1.5	1.6	0.6	7.3	7.8	44.7

Supplementary Table 5. Summary of N fluxes (denitrification and leaching N losses, N sinks, and N inputs) and f_{denit} simulated by the CMIP6 Earth System Models (ESMs).

	Denit. N loss	Leaching N loss	Total N loss	N sink	N input	f_{denit}
AWI-ESM-1-1-LR	48	16	64	44	109	0.75
CESM2	81	19	100	22	121	0.81
CESM2-FV2	84	15	99	28	127	0.85
CESM2-WACCM	81	19	100	23	123	0.81
CESM2-WACCM-FV2	71	13	84	24	108	0.84
EC-Earth3-Veg	18	36	54	25	79	0.33
EC-Earth3-Veg-LR	16	36	53	24	77	0.31
MIROC-ES2L	136	19	154	18	173	0.88
MPI-ESM-1-2-HAM	79	31	111	20	131	0.72
MPI-ESM1-2-LR	78	31	109	19	128	0.72
NorESM2-LM	81	19	100	22	122	0.81
NorESM2-MM	80	17	97	32	129	0.83
UKESM1-0-LL	90	6	97	21	117	0.94
Mean	73	21	94	25	119	0.74
SD	31	9	27	7	24	0.20

Supplementary Table 6. The denitrification N loss (Tg N yr^{-1}) estimated using isotope-benchmarking based f_{denit} and total N losses simulated by the CMIP6 Earth System Models (ESMs).

	The isotope-benchmarking based f_{denit} with N inputs from						Mean	SD
N deposition +BNF	Tian+A	Tian+B	Tian+C	EMEP+A	EMEP+B	EMEP+C		
AWI-ESM-1-1-LR	28	28	30	28	28	30	28	1
CESM2	37	37	41	36	36	41	38	2
CESM2-FV2	37	37	41	36	36	40	38	2
CESM2-WACCM	38	37	41	37	36	41	38	2
CESM2-WACCM-FV2	32	31	35	31	31	35	32	2
EC-Earth3-Veg	22	22	24	22	22	24	23	1
EC-Earth3-Veg-LR	22	22	24	21	21	23	22	1
MIROC-ES2L	62	62	67	61	61	67	63	3
MPI-ESM-1-2-HAM	46	46	50	46	45	50	47	2
MPI-ESM1-2-LR	45	45	49	45	44	49	46	2
NorESM2-LM	38	37	41	37	36	41	38	2
NorESM2-MM	38	37	41	37	37	40	38	2
UKESM1-0-LL	39	39	43	39	39	43	40	2
Mean	37	37	41	37	36	40	38	
SD	11	10	12	10	10	11		11

Supplementary Table 7. Predictors and their data sources for predicting the global soil $\delta^{15}N$ map.

Variables	Long-name	Datasets	Period	Resolution (°)
BD	Bulk density	BNU	recent decades	1/120
pH	Soil pH	BNU	recent decades	1/120
Clay	Fraction of clay in 0-30cm	BNU	recent decades	1/120
Sand	Fraction of sand in 0-30cm	BNU	recent decades	1/120
Silt	Fraction of silt in 0-30cm	BNU	recent decades	1/120
OC	Organic C in 0-30cm	BNU	recent decades	1/120
C/N	Soil C:N ratio in 0-30cm	BNU	recent decades	1/120
AM	Arbuscular mycorrhizal fungi	Steidinger *et al.*[31]	recent decades	1
ECM	Ectomycorrhizal fungi	Steidinger *et al.*[31]	recent decades	1
Nfix	Nitrogen fixing bacteria	Steidinger *et al.*[31]	recent decades	1
GPP	Gross primary production	Keenan *et al.*[22]	1982-2015	0.5
NHx	Monthly NHx deposition	Tian et al.[3] & EMEP[4]	1981-2016 & 2010	0.5
Noy	Monthly Noy deposition	Tian et al.[3] & EMEP[4]	1981-2016 & 2010	0.5
P	Mean annual precipitation	CRU TS v4.03	1981-2018	0.5
P/ET	Mean annual aridity index	CRU TS v4.03	1981-2018	0.5
T	Mean annual temperature	CRU TS v4.03	1981-2018	0.5

Supplementary Table 8. The changes in the isotope-based f_{denit} and in the f_{denit} simulated by Earth System Models (ESMs) under four assumed scenarios of NH_3 volatilization. S1 and S2 represent the scenarios where NH_3 volatilization accounts for 1% and 5%, respectively, of total N loss in grid cells with >50% of the area covered by crops and pasture; S3 and S4 represent the scenarios where NH_3 volatilization accounts for 1% and 3%, respectively, of total N losses in tropical grid cells (25°S–25°N).

	S1	S2	S3	S4
Isotope-based f_{denit}	-0.002	-0.012	-0.010	-0.029
Mean of ESMs' f_{denit}	-0.0003	-0.0015	-0.0007	-0.0022
AWI-ESM-1-1-LR	-0.0001	-0.0007	-0.0006	-0.0018
CESM2	-0.0003	-0.0014	-0.0005	-0.0014
CESM2-FV2	-0.0002	-0.0013	-0.0004	-0.0013
CESM2-WACCM	-0.0003	-0.0014	-0.0004	-0.0013
CESM2-WACCM-FV2	-0.0002	-0.0012	-0.0004	-0.0013
EC-Earth3-Veg	-0.0007	-0.0038	-0.0020	-0.0062
EC-Earth3-Veg-LR	-0.0007	-0.0039	-0.0021	-0.0065
MIROC-ES2L	-0.0001	-0.0007	-0.0004	-0.0013
MPI-ESM-1-2-HAM	-0.0002	-0.0009	-0.0007	-0.0020
MPI-ESM1-2-LR	-0.0002	-0.0009	-0.0007	-0.0020
NorESM2-LM	-0.0003	-0.0015	-0.0005	-0.0014
NorESM2-MM	-0.0003	-0.0014	-0.0004	-0.0013
UKESM1-0-LL	-0.0000	-0.0001	-0.0002	-0.0005

Supplementary Table 9. Impacts of NH_3 volatilization on the steady-state estimates of denitrification N losses. The denitrification N loss is estimated as the product of N input (130 Tg N yr^{-1}, one out of the six sets of N input) and the isotope-based f_{denit}. S1 and S2 represent scenarios where NH_3 volatilization accounts for 1% and 5%, respectively, of total N loss in grid cells with >50% of the area covered by crops and pasture; S3 and S4 represent the scenarios where NH_3 volatilization accounts for 1% and 3%, respectively, of total N losses in tropical grid cells (25°S–25°N).

	Denitrification N losses (Tg N yr^{-1})	
	Mean	SD
Control	56	2
S1	56	2
S2	55	2
S3	55	2
S4	52	2

Supplementary Table 10. The $\delta^{15}N$ signals for different types of rock obtained from Holloway and Dahlgren[32].

Rock categories	Lower Bound (‰)	Upper Bound (‰)	Mean (‰)
Igneous	2.86	9.23	6.05
Sedimentary	0.61	4.94	2.77
Metasedimentary	3.62	7.43	5.53
Hydro and Deposit	7.10	13.51	10.30
Precambrian	-3.14	11.47	4.17
Mean	2.21	9.32	5.76

Supplementary Table 11. Summary of the most widely used fractionation factors and our adopted values in the derivation of the isotope-based f_{denit}.

	Deposition	BNF	Rock N weathering	Denitrification	Nitrification	Leaching
Mariotti *et al.*[18]				20–30		
Robinson[21]		0–6		28–33	35–60	
Houlton and Bai[16]	-3–1	-2–0		5–30		-0.8
Bai et al.[17]	-3–3	-2–0		[3.4–20]		[0–1]
Bai et al.[13]	-1.5	0		[16–20]		[0–5]
Kritee et al.[33]				10–15		
Vitousek et al.[34]	0–1	-2–0	2	15		0
Houlton et al.[5]	0	-1.3		10–20		0.8
Craine et al.[35]		0–2.5	-11–24	0–40		0
Denk et al.[12]		-2±2.2		14.0–31.3	29.6	
Holloway and Dahlgren[32]			Supplementary Table 10			
Our adopted value	0	-2	Supplementary Table 10	13 (10–20)	/	0

Supplementary Table 12. Detailed information on the outputs of Earth System Models (ESMs) in CMIP6 extracted for our analysis.

Source ID	Land surface model	Experiment	Variant	Resolution
ACCESS-ESM1-5	CABLE2.4	Historical	r1i1p1f1	250 km
AWI-ESM-1-1-LR	CLM4.5	Historical	r1i1p1f1	100 km
CESM2	CLM5	Historical, 1pctCO2, 1pctCO2-bgc	r10i1p1f1	100 km
CESM2-FV2	CLM5	Historical	r1i1p1f1	250 km
CESM2-WACCM	CLM5	Historical	r1i1p1f1	100 km
CESM2-WACCM-FV2	CLM5	Historical	r1i1p1f1	250 km
EC-Earth3-Veg	LPJ-GUESS v4	Historical	r5i1p1f1/r4i1p1f1	100 km
EC-Earth3-CC	LPJ-GUESS v4	Historical	r1i1p1f1	100 km
EC-Earth3-Veg-LR	LPJ-GUESS v4	Historical	r1i1p1f1	250 km
MIROC-ES2L	MATSIRO6.0+VISIT-e ver.1.0	Historical, 1pctCO2, 1pctCO2-bgc	r1i1p1f2	500 km
MPI-ESM-1-2-HAM	JSBACH 3.20	Historical	r1i1p1f1	250 km
MPI-ESM1-2-LR	JSBACH3.20	Historical	r1i1p1f1	250 km
NorESM2-LM	CLM5	Historical, 1pctCO2, 1pctCO2-bgc	r1i1p1f1	250 km
NorESM2-MM	CLM5	Historical, 1pctCO2, 1pctCO2-bgc	r1i1p1f1	100 km
UKESM1-0-LL	JULES-ES-1.0	Historical, 1pctCO2, 1pctCO2-bgc	r1i1p1f2	100 km

Supplementary References

1. Craine, J. M. *et al.* Convergence of soil nitrogen isotopes across global climate gradients. *Sci. Rep.*, **5,** 8280 (2015).

2. Sena-Souza, J. P., Houlton, B. Z., Martinelli, L. A. & Bielefeld Nardoto, G. Reconstructing continental-scale variation in soil $\delta^{15}N$: a machine learning approach in South America. *Ecosphere*, **11**, e03223 (2020).

3. Tian, H. *et al.* The global N_2O model intercomparison project. *Bull. Am. Meteorol. Soc.*, **99**, 1231-1251 (2018).

4. Schwede, D. B. et al. Spatial variation of modelled total, dry and wet nitrogen deposition to forests at global scale. *Environ. Pollut.*, **243**, 1287-1301 (2018).

5. Houlton, B. Z., Marklein, A. R. & Bai, E. Representation of nitrogen in climate change forecasts. *Nature Clim. Change*, **5**, 398-401 (2015).

6. Amundson, R. *et al.* Global patterns of the isotopic composition of soil and plant nitrogen. *Global Biogeochem. Cycles*, **17**, 1031 (2003).

7. Houlton, B. Z., Morford, S. L., Dahlgren, R. A. Convergent evidence for widespread rock nitrogen sources in Earth's surface environment. *Science*, **360**, 58-62 (2018).

8. Peng, J., Wang, Y.-P., Houlton, B. Z., Dan, L., Pak, B. & Tang, X. Global carbon sequestration is highly sensitive to model-based formulations of nitrogen fixation. *Global Biogeochem. Cycles*, **34**, e2019GB006296 (2020).

9. Goll, D. S. *et al.* Carbon–nitrogen interactions in idealized simulations with JSBACH (version 3.10). *Geosci. Model Dev.*, **10**, 2009-2030 (2017).

10. Wang, A. *et al.* High nitrogen isotope fractionation of nitrate during denitrification in four forest soils and its implications for denitrification rate estimates. *Sci. Total Environ.*, **633,** 1078-1088 (2018).

11. Su C. X. *et al.* $\delta^{15}N$ of nitric oxide produced under aerobic or anaerobic conditions from seven soils and their associated N isotope fractionations. *J. Geophys. Res. Biogeosci.*, **125**, e2020JG005705 (2020).

12. Denk, T. R. A. *et al.* The nitrogen cycle: A review of isotope effects and isotope modeling approaches. *Soil Biol. Biochem.*, **105,** 121-137 (2017).

13. Bai, E., Houlton, B. Z., Wang, Y. P. Isotopic identification of nitrogen hotspots across natural terrestrial ecosystems. *Biogeosciences*, **9**, 3287-3304 (2012).

14. Craine, J. M. et al. Ecological interpretations of nitrogen isotope ratios of terrestrial plants and soils. Plant Soil, 396, 1-26 (2015).

15. Houlton, B. Z., Sigman, D. M. & Hedin, L. O. Isotopic evidence for large gaseous nitrogen losses from tropical rainforests. *Proc. Natl. Acad. Sci. U. S. A.*, **103**, 8745-8750 (2006).

16. Houlton, B. Z. & Bai, E. Imprint of denitrifying bacteria on the global terrestrial biosphere. *Proc. Natl. Acad. Sci. U. S. A.*, **106**, 21713-21716 (2009).

17. Bai, E. & Houlton, B. Z. Coupled isotopic and process-based modeling of gaseous nitrogen losses from tropical rain forests. *Global Biogeochem. Cycles*, **23**, GB2011 (2009).

18. Mariotti, A. *et al.* Experimental-determination of nitrogen kinetic isotope fractionation: Some principles; illustration for the denitrification and nitrification processes. Plant Soil, 62, 413-430 (1981).

19. Sutton, M. A. et al. Towards a climate-dependent paradigm of ammonia emission and deposition. Philos. Trans. R. Soc. Lond. B, Biol. Sci., **368**, 20130166 (2013).

20. Fowler, D. *et al.* The global nitrogen cycle in the twenty-first century. *Philos. Trans. R. Soc. Lond. B, Biol. Sci.*, **368**, 20130164 (2013).

21. Robinson, D. δ^{15}N as an integrator of the nitrogen cycle. *Trends Ecol. Evol.*, **16**, 153-162 (2001).

22. Keenan, T. F. *et al.* Recent pause in the growth rate of atmospheric CO_2 due to enhanced terrestrial carbon uptake. *Nat. Commun.*, **7,** 13428 (2016).

23. Moreno-Martínez, A. et al. A methodology to derive global maps of leaf traits using remote sensing and climate data, Remote Sens. Environ., **218**, 69-88 (2018).

24. Deng, M. *et al.* Ecosystem scale trade-off in nitrogen acquisition pathways. *Nat. Ecol. Evol.,* **2**, 1724–1734 (2018).

25. Shi, M., Fisher, J. B., Brzostek, E. R. & Phillips, R. P. Carbon cost of plant nitrogen acquisition: global carbon cycle impact from an improved plant nitrogen cycle in the Community Land Model. *Glob. Chang. Biol.*, **22**, 1299-1314 (2016).

26. Dürr, H. H. , Meybeck, M. & Dürr, S. H. Lithologic composition of the Earth's continental surfaces derived from a new digital map emphasizing riverine material transfer. *Global Biogeochemical Cycles*, **19**, GB4S10 (2005).

27. Wang, Y. P., Law, R. M. & Pak, B.. A global model of carbon, nitrogen and phosphorus cycles for the terrestrial biosphere. *Biogeosciences*, **7**, 2261-2282 (2010).

28. Smith, B. *et al.* Implications of incorporating N cycling and N limitations on primary production in an individual-based dynamic vegetation model. *Biogeosciences*, **11**, 2027-2054 (2014).

29. Inatomi, M., Ito, A., Ishijima, K. & Murayama, S. Greenhouse gas budget of a cool-temperate deciduous broad-leaved forest in Japan estimated using a process-based model. *Ecosystems*, **13**, 472-483 (2010).

30. Wiltshire, A. J. et al. JULES-CN: a coupled terrestrial carbon–nitrogen scheme (JULES vn5.1). Geosc. Model Dev., **14**, 2161-2186 (2021).

31. Steidinger, B. S. *et al.* Climatic controls of decomposition drive the global biogeography of forest-tree symbioses. *Nature*, **569**, 404-408 (2019).

32. Holloway, J. M. & Dahlgren, R. A. Nitrogen in rock: Occurrences and biogeochemical implications. Global Biogeochem. Cycles, **16**, 1118 (2002).

33. Kritee, K. *et al.* , Reduced isotope fractionation by denitrification under conditions relevant to the ocean, *Geochim. Cosmochim. Acta*, **92**, 243–259 (2012).

34. Vitousek, P. M., Menge, D. N., Reed, S. C. & Cleveland, C. C. Biological nitrogen fixation: rates, patterns and ecological controls in terrestrial ecosystems. *Philos. Trans. R. Soc. Lond. B, Biol. Sci.*, **368**, 20130119 (2013).

35. Craine, J. M. *et al.* Ecological interpretations of nitrogen isotope ratios of terrestrial plants and soils. *Plant Soil*, **396**, 1-26 (2015).
